# A novel chemical genetic approach reveals paralog-specific role of ERK1/2 in mouse embryonic stem cell fate control

**DOI:** 10.3389/fcell.2024.1415621

**Published:** 2024-07-12

**Authors:** Liang Hu, Xiong Xiao, Wesley Huang, Tao Zhou, Weilu Chen, Chao Zhang, Qi-Long Ying

**Affiliations:** ^1^ Department of Stem Cell Biology and Regenerative Medicine, Eli and Edythe Broad Center for Regenerative Medicine and Stem Cell Research at USC, Keck School of Medicine, University of Southern California, Los Angeles, CA, United States; ^2^ Loker Hydrocarbon Research Institute and Department of Chemistry, University of Southern California, Los Angeles, CA, United States

**Keywords:** chemical genetics, embryonic stem cells, self-renewal, differentiation, MAPK pathways, ERK1/2 (p44/42 MAPK), paralog-specific inhibition

## Abstract

**Introduction:** Mouse embryonic stem cell (ESC) self-renewal can be maintained through dual inhibition of GSK3 and MEK kinases. MEK has two highly homologous downstream kinases, extracellular signal-regulated kinase 1 and 2 (ERK1/2). However, the exact roles of ERK1/2 in mouse ESC self-renewal and differentiation remain unclear.

**Methods:** We selectively deleted or inhibited ERK1, ERK2, or both using genetic and chemical genetic approaches combined with small molecule inhibitors. The effects of ERK paralog-specific inhibition on mouse ESC self-renewal and differentiation were then assessed.

**Results:** ERK1/2 were found to be dispensable for mouse ESC survival and self-renewal. The inhibition of both ERK paralogs, in conjunction with GSK3 inhibition, was sufficient to maintain mouse ESC self-renewal. In contrast, selective deletion or inhibition of only one ERK paralog did not mimic the effect of MEK inhibition in promoting mouse ESC self-renewal. Regarding ESC differentiation, inhibition of ERK1/2 prevented mesendoderm differentiation. Additionally, selective inhibition of ERK1, but not ERK2, promoted mesendoderm differentiation.

**Discussion:** These findings suggest that ERK1 and ERK2 have both overlapping and distinct roles in regulating ESC self-renewal and differentiation. This study provides new insights into the molecular mechanisms of ERK1/2 in governing ESC maintenance and lineage commitment, potentially informing future strategies for controlling stem cell fate in research and therapeutic applications.

## 1 Introduction

The Mitogen-Activated Protein Kinase (MAPK) signaling pathway plays a crucial role in various cellular processes, encompassing proliferation, differentiation, cell survival, migration, among others (Yang et al., 2019). Upon receiving mitogenic stimuli, cells activate the MAPK pathway, initiating a kinase cascade involving RAF-MEK-ERK. Extracellular regulated kinase (ERK) serves as the effector kinase of the MAPK/ERK pathway. In mammals, ERK1 and ERK2 are two highly homologous paralogs. Despite sharing approximately 85% of their overall amino acid sequence, ERK1/2 have long been regarded as functionally redundant proteins (Fremin et al., 2015; Erdenebaatar et al., 2023). However, numerous studies have demonstrated that ERK1/2 exhibit distinct functionalities, particularly in development and cancer biology (Busca et al., 2016; Gagliardi et al., 2020; Guegan et al., 2013; Ricard et al., 2019; Vantaggiato et al., 2006; von Thun et al., 2012). For example, while *Erk1* ^−/−^ mouse embryos are viable and fertile, *Erk2* ^−/−^ mice experience embryonic lethality due to placental defects (Pages et al., 1999; Hatano et al., 2003; Saba-El-Leil et al., 2003). Nevertheless, the concept of functional divergence between the two ERK paralogs remains debatable (Busca et al., 2016).

Deciphering the paralog-specific functions of ERK in diverse cellular processes presents a significant challenge. To date, no small molecules have been identified that can selectively target one ERK paralog over the other. While genetic knockout and knockdown techniques offer powerful means to characterize kinase functions, they not only inhibit kinase activity but also disrupt scaffold and protein-protein interactions, potentially complicating data interpretation (Chen et al., 2017). A chemical genetic approach known as the bump-hole method has been developed to elucidate functional differences between highly homologous kinases (Zhang et al., 2005). This method involves generating an analog-sensitive mutant by introducing a mutation at the gatekeeper position, enabling specific inhibition of the engineered kinase without affecting its wild-type homolog.

Embryonic stem cells (ESCs) possess the remarkable ability to indefinitely maintain their undifferentiated state and differentiate into nearly every cell type in the body, making them invaluable *in vitro* cell sources for cell therapy, drug screening, and developmental studies (Smith, 2001). Inhibition of the MAPK/ERK pathway using a MEK inhibitor, PD0325901 (PD), has been shown to promote ESC self-renewal by blocking the differentiation program (Ying et al., 2008). Combining PD with a GSK3 inhibitor (CHIR99021, CHIR) robustly propagates ESCs with high germ-line transmission efficiency (Ying et al., 2008; Blair et al., 2011; Chen et al., 2015; Ying and Smith, 2017). Notably, ERK1/2 were traditionally regarded as the sole substrates of MEK kinases until 2015 (Tang et al., 2015). However, it remains unknown whether the inhibition of ERK1/2 can substitute for the role of PD in maintaining ESCs in the presence of CHIR. Additionally, the specific roles of individual ERK1/2 paralogs in ESC self-renewal and differentiation have yet to be defined.

Here, we generated ERK1/2 double knockout and single knockout ESC lines to investigate the distinct roles of the highly homologous ERK1/2 paralogs in ESC fate control. Additionally, we developed a novel inhibitor-resistant approach to selectively inhibit one of the ERK1/2 paralogs in ESCs. By combining knockout and inhibitor-resistant approaches in ESCs, we delineated the roles of individual ERK paralogs in ESC self-renewal and differentiation, revealing both overlapping and non-overlapping functions of ERK1/2 in ESC self-renewal and differentiation, respectively. This study paves the way for further exploration of the roles of individual ERK paralogs in different cell types and during various cellular processes.

## 2 Results

### 2.1 Establishment and characterization of *Erk1/2* ^−/−^ ESCs

**FIGURE 1:**
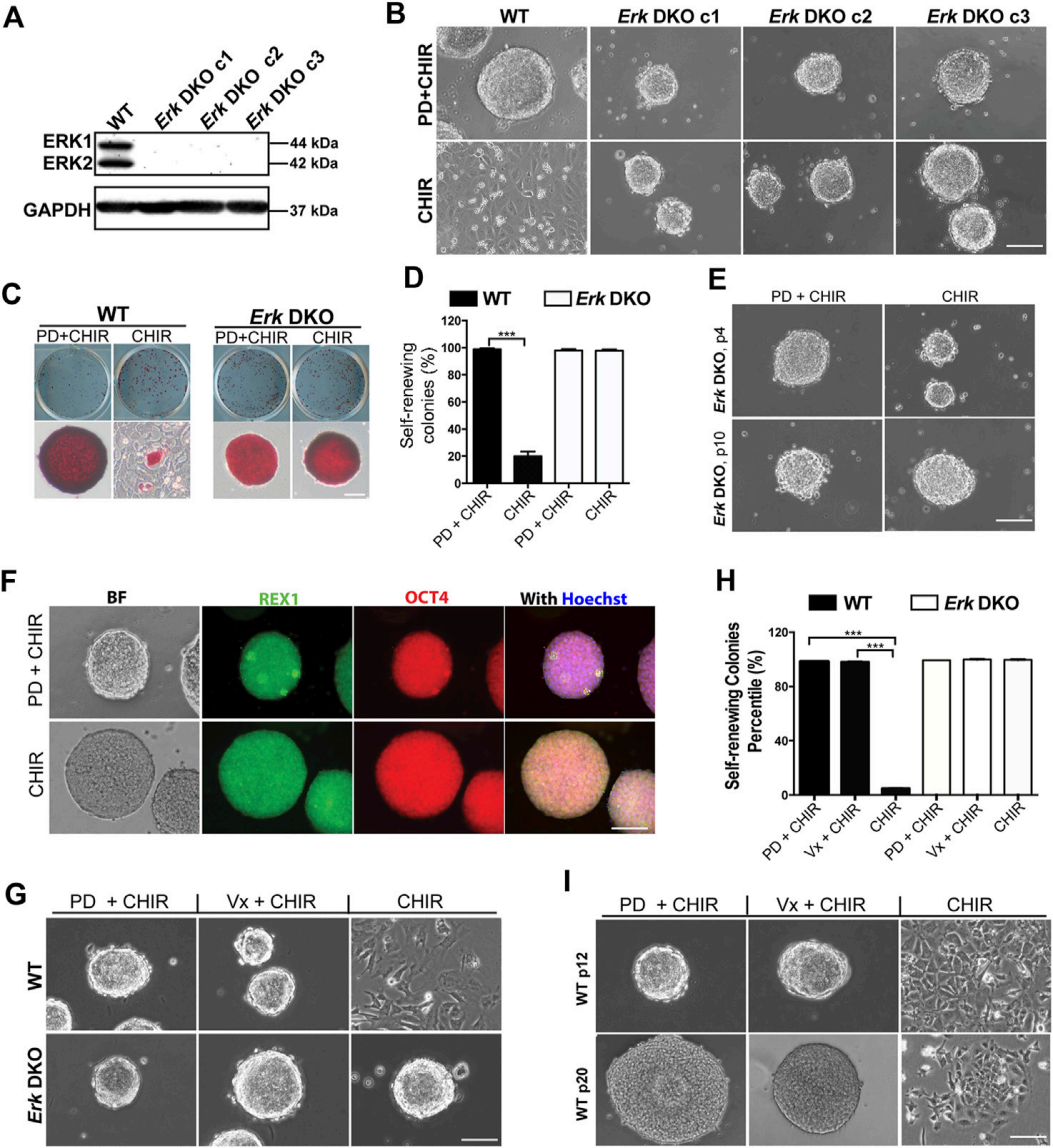
Inhibition of both ERK1/2 in ESCs is sufficient for the maintenance of mouse ESCs in the presence of CHIR. **(A)** Western blot analysis of the expression of ERK1/2 and GAPDH in wild-type (WT) and *Erk* DKO ESCs. ERK1: 44 kDa, ERK2: 42 kDa. GAPDH: 37 kDa. **(B)** Representative phase contrast images of WT ESCs and different clones of *Erk* DKO ESCs cultured under the indicated conditions in serum-free N2B27 medium. **(C)** Representative images of colonies formed from the indicated ESC lines cultured under PD03 + CHIR or CHIR alone condition for 7 days. ESCs were plated onto 12-well plates and cultured under the indicated conditions. Undifferentiated ESCs were stained in red, termed as AP+; differentiated ESCs could not be stained, named as AP-. **(D)** Quantification of the percentages of self-renewing colonies (100% AP + ESCs) formed in WT ESCs and *Erk* DKO ESCs as shown in **(C)**. Data represent means ± SEM of three biological replicates (n = 3). ***: *p* < 0.001. **(E)** Representative phase contrast images of *Erk* DKO ESCs cultured in N2B27 medium under indicated conditions. The result of one representative clone is shown here. p: passage. **(F)** Representative images of immunofluorescence staining of REX1 and OCT4 in WT and *Erk* DKO ESCs. **(G)** Representative phase contrast images of WT ESCs and *Erk* DKO ESCs cultured under the indicated conditions. **(H)** Quantification of the percentages of self-renewing colonies formed in WT ESCs and *Erk* DKO ESCs as shown in **(G)**. Data represent means ± SEM (n = 3). **(I)** Representative phase contrast images of continuously passaged WT ESCs (p12 and p20) cultured in N2B27 medium supplemented with CHIR, Vx11e + CHIR, or PD + CHIR. Scale bars, 100 µm.

To elucidate the roles of ERK1/2 in ESCs, we initially generated *Erk1/2* double knockout (*Erk* DKO) ESCs using CRISPR/Cas9 technology. The ESC line utilized was the 46C ESC line derived from the 129 strain of mouse (Ying et al., 2003). Thirty-three clones were selected from the pool of candidate *Erk* DKO ESCs, out of which seven exhibited complete deletion of Erk1/2. Three clones of *Erk* DKO ESC lines confirmed by immunoblotting (Figure 1A) and DNA sequencing (Supplementary Figure S1A) were selected for further investigation.

We first characterized the survival and genome stability of these *Erk* DKO ESCs. In the presence of LIF and serum (LIF + serum), all three clones of *Erk* DKO ESCs remained viable and exhibited a flat morphology similar to wild-type (WT) ESCs. However, *Erk DKO ESCs* appeared more uniform with fewer differentiated cells of irregular sizes compared to WT ESCs, indicating a more undifferentiated state of *Erk* DKO ESCs (Supplementary Figure S1B). Upon continuous passaging for ten passages in LIF + serum, these cells maintained viability and normal cell morphology (Supplementary Figure S1C). Karyotyping of these long-term cultured *Erk* DKO ESCs showed that the majority had a normal chromosome number, suggesting genome stability in the absence of ERK1/2 (Supplementary Figure S1D).

It is well-established that inhibition of MAPK/ERK signaling promotes ESC self-renewal, while its activation primes ESC differentiation (Burdon et al., 2002; Lanner and Rossant, 2010). Consequently, we investigated the effects of ERK1/2 ablation on ESC self-renewal and differentiation, two fundamental features of pluripotent stem cells. Regarding self-renewal ability, the expression of naive pluripotency markers (*Rex1*, *Nanog*, and *Oct4*) was profiled by real-time quantitative PCR (qPCR) in *Erk* DKO ESCs. *Erk* DKO ESCs exhibited a similar level of Oct4 but higher levels of *Rex1* and *Nanog* compared to WT ESCs (Supplementary Figure S1E). Consistent with this, *Erk* DKO ESCs demonstrated significantly lower percentages of Nanog-negative cells by immunostaining, indicating enhanced self-renewal following ERK1/2 ablation (Supplementary Figure S1F).

To study ERK1/2’s role in ESC differentiation, we conducted embryoid body (EB) differentiation assays in *Erk* DKO ESCs. A 4-/4+ retinoic acid-based EB differentiation protocol was employed. qPCR analysis was performed on one naive pluripotency marker (*Rex1*) and three lineage-specific markers (*Sox1* for neuroectoderm, *Brachyury* for early mesendoderm, and *FoxA2* for endoderm) to assess differentiation status. On both day 4 and day 8, *Erk* DKO ESC-derived EBs exhibited significantly higher levels of *Rex1* but significantly lower levels of the three lineage markers compared to WT ESC-derived EBs (Supplementary Figure S1G), indicating a slower rate of differentiation in *Erk* DKO ESCs.

To confirm the differentiation potential of *Erk* DKO ESCs further, day 8 EBs were collected and re-plated to induce differentiation into mature cell types of different germ layers. WT ESCs readily differentiated into mature cells of both neuroectoderm and mesendoderm lineages, as evidenced by strongly positive staining of lineage-specific markers (Supplementary Figure S1H). In contrast, while *Erk* DKO ESCs could differentiate into TUJ1^+^ (neuronal lineage marker) cells, they showed no cells positive for mesendodermal markers (Myosin or GATA4) (Supplementary Figure S1H), indicating preserved ability for neuroectodermal differentiation but loss of ability for mesendodermal differentiation in *Erk* DKO ESCs. Overall, these findings suggest that ERK1/2 are dispensable for mouse ESC survival. Their deletion enhances self-renewal but impairs differentiation into mesodermal and endodermal cells.

### 2.2 Deletion or chemical inhibition of ERK1/2 sustains ESC self-renewal in the presence of GSK3 inhibitor

Dual inhibition of GSK3 and MEK maintains mouse ESCs undifferentiated in the long term (Ying et al., 2008; Ying and Smith, 2017). To determine whether the deletion of ERK1/2 in ESCs could mimic the effects of MEK inhibition by PD, we cultured WT ESCs and *Erk* DKO ESCs in serum-free N2B27 medium supplemented with PD + CHIR (2i) or CHIR alone. WT ESCs remained undifferentiated and formed compact colonies with sharp boundaries in the 2i medium; however, the majority of WT ESCs differentiated and became flat in the CHIR alone medium (Figure 1B). In contrast, *Erk* DKO ESCs formed uniformly compact colonies under both the 2i and the CHIR alone conditions (Figure 1B).

To assess the self-renewal status of WT ESCs and *Erk* DKO ESCs maintained in 2i or the CHIR alone condition, we performed alkaline phosphatase (AP) staining. In the AP staining assay, undifferentiated ESCs are stained red (termed AP+); in contrast, differentiated ESCs cannot be stained (termed AP-) due to the loss of alkaline phosphatase over the course of differentiation (Figure 1C). The dome-shaped colonies consisting of AP + cells are classified as self-renewing colonies, and the colonies with AP- cells and rough boundaries are termed as differentiated colonies. For WT ESCs, the percentage of self-renewing colonies in the CHIR alone medium was significantly lower than that in the 2i medium (Figure 1D), suggesting that WT ESCs underwent differentiation under the CHIR alone condition. In contrast, for the *Erk* DKO ESCs, percentages of self-renewing colonies under the 2i condition were similar to those under the CHIR alone condition (Figure 1D). This result suggests that *Erk* DKO ESCs may be able to self-renew under the CHIR alone condition for the long term. To test this hypothesis, we continuously passaged *Erk* DKO ESCs for more than ten passages. We found that *Erk* DKO ESCs remained undifferentiated in the presence of CHIR alone based on the colony morphology (Figure 1E) and the expression of pluripotency markers REX1 and OCT4 (Figure 1F).

To further confirm the role of ERK1/2 in ESC self-renewal and differentiation, we tested an ERK1/2 inhibitor (Vx11e) on ESCs to see if it could replace the role of PD in the 2i condition to maintain ESC self-renewal. We combined Vx11e with CHIR and tested this new combination in both WT ESCs and *Erk* DKO ESCs. These cells exhibited compact colonies under both the 2i and the Vx11e + CHIR conditions (Figure 1G). Consistent with the above results, in the AP staining assay, both WT ESCs and *Erk* DKO ESCs cultured in the Vx11e + CHIR medium had very high percentages of self-renewing colonies, comparable to those percentages in these two types of ESCs cultured in the 2i medium (Figure 1H). To further evaluate the effect of Vx11e + CHIR on ESC self-renewal in the long term, we continuously passaged WT ESCs and cultured them under this condition for over 20 passages. The results showed that WT ESCs could always form uniform compact colonies under both the 2i and the Vx11e + CHIR conditions (Figure 1I). These results indicate that the inhibition of kinase activity of ERK1/2 by Vx11e can fully recapitulate the effect of PD in maintaining ESC self-renewal in the presence of CHIR.

In summary, our data suggest that inhibition of ERK1/2 by either knockout or a chemical inhibitor is sufficient to maintain ESC self-renewal together with GSK3 inhibition.

### 2.3 Deletion of either ERK1 or ERK2 is insufficient for ESC self-renewal in the presence of CHIR

**FIGURE 2:**
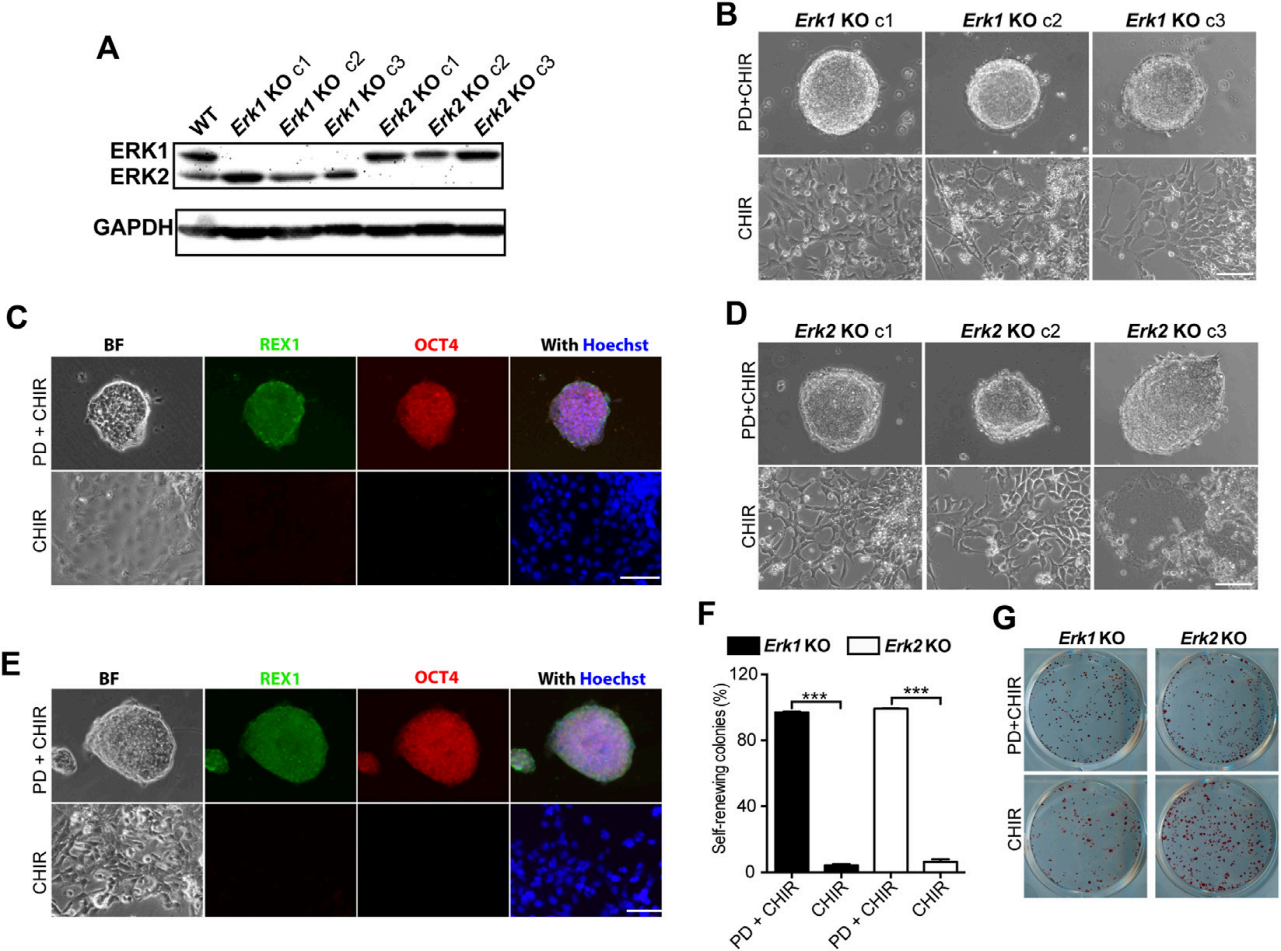
Knocking-out ERK1 or ERK2 is not sufficient to maintain ESC self-renewal in the presence of CHIR. **(A)** Western blot analysis of the expression of ERK1/2 and GAPDH in WT, *Erk1* ^−/−^, and *Erk2* ^−/−^ ESCs. **(B)** Representative phase contrast images of different clones of *Erk1* ^−/−^ ESCs cultured under indicated conditions in serum-free N2B27 medium. **(C)** Representative images of immunofluorescence staining of REX1 and OCT4 in *Erk1* ^−/−^ ESCs. **(D)** Representative phase contrast images of different clones of *Erk2* ^*−/−*^ ESCs cultured under indicated conditions in serum-free N2B27 medium. **(E)** Representative images of immunofluorescence staining of REX1 and OCT4 in *Erk2* ^−/−^ ESCs. **(F)** Quantification of the percentages of self-renewing and differentiated colonies after AP staining in *Erk1* ^−/−^ ESCs and *Erk2* ^−/−^ ESCs cultured in N2B27 medium supplemented with CHIR or PD + CHIR. Data represent means ± SEM (n = 3). ***: *p* < 0.001. **(G)** Representative AP-staining images of colonies formed from the indicated ESC lines cultured under PD03 + CHIR or CHIR alone condition for 7 days. Scale bars, 100 µm.

To determine the roles of individual ERK isoforms in ESCs, *Erk1* knockout ESCs (*Erk1* ^*−/−*^ ESCs) and *Erk2* knockout ESCs (*Erk2* ^*−/−*^ ESCs) were generated using CRISPR technology. Upon screening via immunoblotting, nine out of the twenty picked clones in the pool of candidate *Erk1* ^*−/−*^ ESCs showed the deletion of ERK1, while eight out of the twenty-two picked clones in the pool of candidate *Erk2* ^*−/−*^ ESCs showed the deletion of ERK2. Three clones each of the *Erk1* ^*−/−*^ ESCs (c1, c2, and c3) and the *Erk2* ^*−/−*^ ESCs (c1, c2, and c3) were selected for further study. These clones were confirmed by both immunoblotting (Figure 2A) and DNA sequencing.

To explore the effects of individual ERK isoform deletion on ESC self-renewal, we cultured *Erk1* ^*−/−*^ ESCs and *Erk2* ^*−/−*^ ESCs under the 2i and CHIR-alone conditions. All three clones of *Erk1* ^*−/−*^ ESCs under the 2i condition formed dome-shaped colonies (Figure 2B) and were positive for REX1 and OCT4 (Figure 2C). However, they differentiated and became flat under the CHIR-alone condition, exhibiting negative staining for REX1 and OCT4 (Figure 2C). Similar results were obtained from the three clones of *Erk2* ^*−/−*^ ESCs (Figures 2D, E). These findings suggest that inhibition of ERK1 in *Erk2* ^*−/−*^ ESCs and inhibition of ERK2 in *Erk1* ^*−/−*^ ESCs by the MEK inhibitor (PD) are necessary to maintain their undifferentiated status under the CHIR-alone condition.

To further confirm the self-renewal status of these ESCs under the CHIR-alone or 2i conditions, we performed AP staining assays and quantified the percentages of self-renewing colonies in *Erk1* ^*−/−*^ ESCs and *Erk2* ^*−/−*^ ESCs. It was revealed that in either *Erk1* ^*−/−*^ ESCs or *Erk2* ^*−/−*^ ESCs, the percentage of self-renewing colonies under the CHIR-alone condition was significantly lower than that under the 2i condition (Figures 2F, G). These results suggest that deletion of one of the ERK1/2 isoforms is not sufficient for ESC self-renewal in the presence of a GSK3 inhibitor.

### 2.4 Designing an inhibitor-resistant approach for ERK paralog-specific inhibition

It is well known that pharmacological inhibition and genetic deletion of kinases can result in very different phenotypes (Bi et al., 2002; Papa et al., 2003; Chen et al., 2017). Therefore, we next investigated the effect of chemically inhibiting one of the ERK paralogs on ESC self-renewal. To achieve specific inhibition of one ERK paralog, an ERK-paralog specific inhibitor should be used. However, currently, there are no small molecules that can selectively inhibit one of the two highly homologous ERK1/2 paralogs while sparing the other.

To address this problem, we first attempted the bump-hole approach on ERK1/2 to achieve selective inhibition of either ERK1 or ERK2. Successful utilization of the bump-hole approach for studying the function of individual kinase isoforms requires the kinase mutant to preserve its kinase activity (Zhang et al., 2005). However, we found that the ERK1 analog-sensitive (AS) mutant, ERK1-Q123G, lost its kinase activity in ESCs (data not shown), consistent with the previous finding regarding ERK1-Q123G via *in vitro* kinase assays (Endo et al., 2006). We further attempted a second-site suppressor strategy (Zhang et al., 2005) on the ERK1-AS mutant, but this strategy failed to rescue ERK1 kinase activity.

**FIGURE 3:**
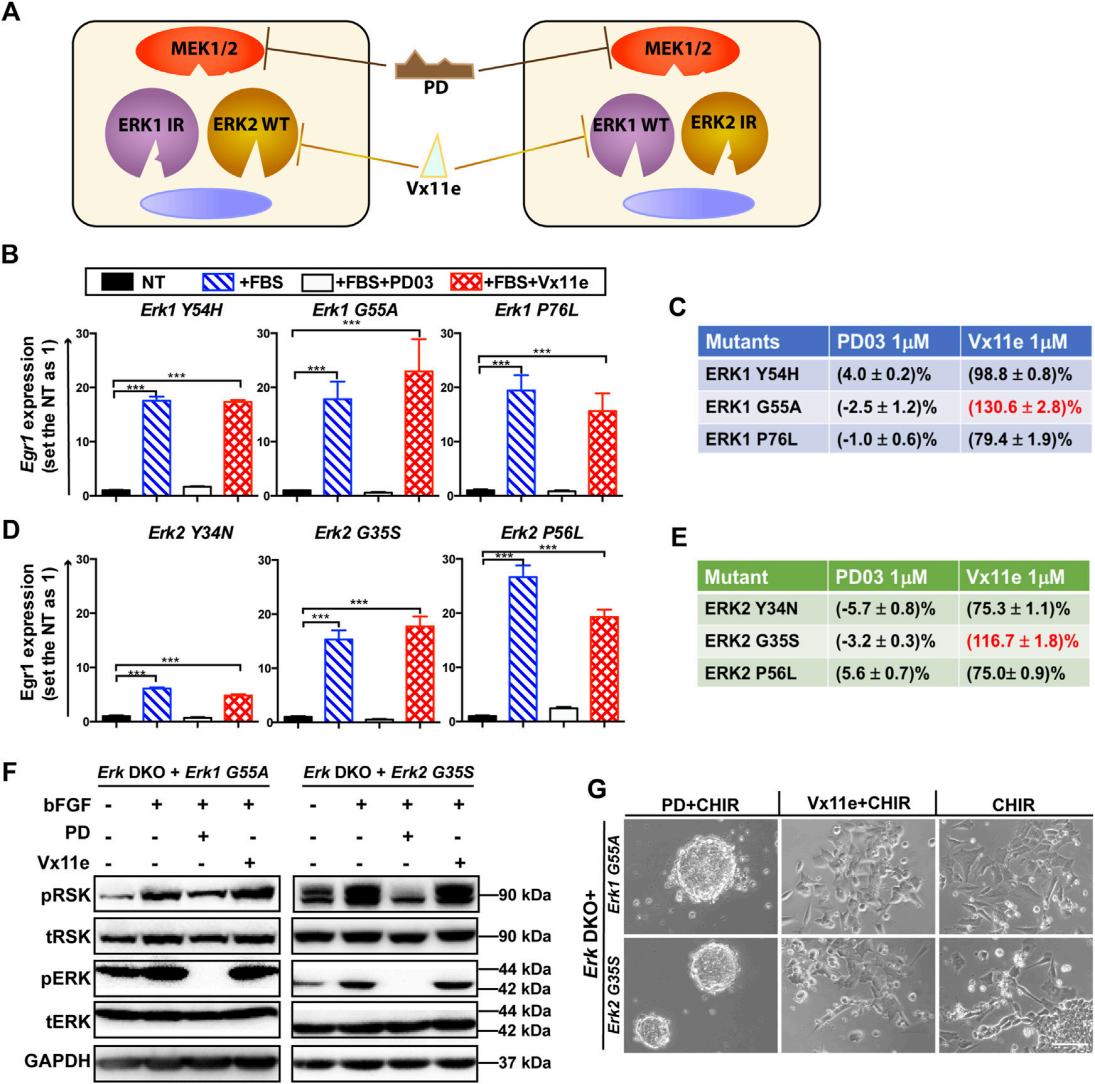
Screening of optimal ERK1/2 IR mutants for the inhibitor-resistant strategy. **(A)** An illustration of the inhibitor-resistant strategy to achieve individual inhibition of highly homologous ERK1/2 paralogs. Left panel: ERK1 IR mutants are expressed in the engineered cells. ERK2 WT proteins are specifically inhibited when Vx11e is added, as ERK1 IR mutants are resistant to Vx11e. Right panel: ERK2 IR mutants are expressed in the engineered cells, and ERK1 WT proteins are selectively inhibited when Vx11e is supplemented. The kinase activity of both ERK1/2 isoforms can be inhibited in both types of cells by PD. IR: inhibitor (Vx11e)-resistant. **(B)** qPCR analyses of *Egr1* levels in the *Erk1* IR transgenic ESC lines (*Erk* DKO + *Erk1* ^Y54H^ ESCs, *Erk* DKO + *Erk1* ^G55A^ ESCs, and *Erk* DKO + *Erk1* ^P76L^ ESCs) under the indicated conditions. The data are shown as mean ± SEM (n = 2). ***: *p* < 0.001. **(C)** The relative kinase activity for the ERK1 IR mutants under the indicated conditions was quantified. For each ERK1 IR transgenic cell line, the NT group was used as a baseline, and the FBS alone group was set as 100%. The relative kinase activities of the PD and Vx11e groups were then calculated. FBS: fetal bovine serum. **(D)** qPCR analyses of *Egr1* levels in the ERK2 IR transgenic ESC lines (*Erk* DKO + *Erk2* ^Y34N^ ESCs, *Erk* DKO + *Erk2* ^G35S^ ESCs, and *Erk* DKO + *Erk2* ^P56L^ ESCs) under the indicated conditions. The data are shown as mean ± SEM (n = 2). ***: *p* < 0.001. **(E)** The relative kinase activity for the ERK2 IR mutants under the indicated conditions was quantified. **(F)** Western blot analysis of the expression of pRSK, tRSK, pERK, tERK, and GAPDH in *Erk1* ^*G55A*^ transgenic ESCs and *Erk2* ^*G35S*^ transgenic ESCs under the indicated conditions. Cells were treated with the indicated combination of bFGF (20 ng/mL), PD, and Vx11e for 10–15 min after overnight serum starvation. bFGF: basic fibroblast growth factor. +: the addition; -: the absence. **(G)** Representative phase contrast images of *Erk1* ^*G55A*^ transgenic ESCs and *Erk2* ^*G35S*^ transgenic ESCs cultured under the indicated conditions in N2B27 medium for 7 days. Scale bars, 100 µm.

We devised a novel inhibitor-resistant (IR) strategy based on an ERK1/2 inhibitor, Vx11e, to achieve selective inhibition of one ERK paralog (Figure 3A). By introducing an IR mutation into one ERK paralog, the mutated ERK paralog will no longer be inhibited by Vx11e. For instance, in *Erk1*-IR knock-in cells, Vx11e can inhibit ERK2 WT proteins but not the ERK1-IR mutant; application of Vx11e will specifically inhibit ERK2 but not ERK1 in these cells.

For the successful application of this IR strategy for studying ERK in ESCs, an ideal ERK-IR mutant should meet at least the following requirements: a) well-preserved kinase activity; b) resistance to Vx11e but not to PD; c) no changes in the studied phenotype(s) when the IR mutant is expressed. Human ERK mutations conferring drug resistance to Vx11e have been revealed *via* drug screening in human cancer cell lines (Goetz et al., 2014). There were three homologous mutations between ERK1 and ERK2. Therefore, we utilized these three homologous mutations to generate ERK-IR mutants in mouse ESCs.

### 2.5 Generation and characterization of optimal ERK mutants for paralog-specific inhibition in ESCs

To identify an optimal ERK mutant for the *Erk*-IR strategy, we screened these three homologous ERK1/2 mutants. Transgenic ESC lines were established, where individually one of the three either ERK1 (Y54H, G55A, P76L) or ERK2 (Y34N, G35S, P56L) homologous mutants was expressed in *Erk* DKO ESCs. In these *Erk*-IR transgenic ESCs (*Erk* DKO + *Erk*-IR ESCs), ERK mutants were co-expressed with RFP linked by a P2A self-cleaving peptide to facilitate tracking of their expression (Supplementary Figure S2A). The expression of these ERK IR mutants and RFP in transgenic ESCs was confirmed by immunoblotting (Supplementary Figure S2B).

A system to evaluate the kinase activity of these ERK mutants was established based on targets induced by active MAPK/ERK signaling. *Egr1* is a downstream target of ERK1/2 and can be induced in both *Erk1* ^*−/−*^ cells and *Erk2* ^*−/−*^ cells (Ye et al., 2013; Lee et al., 2014). We then chose *Egr1* and tested whether its transcriptional levels could represent ERK kinase activity in ESCs. The levels of *Egr1* were significantly increased in the *Erk* WT (*Erk1* WT or *Erk2* WT) transgenic ESCs but not in the RFP transgenic ESCs when these cells were stimulated with serum (Supplementary Figure S2C). In addition, the upregulation of *Egr1* was abolished in the *Erk* WT transgenic ESCs when either PD or Vx11e was supplemented together with serum (Supplementary Figure S2C). These results indicated that the *Egr1* transcriptional level in ESCs is a good readout for ERK kinase activity.

We evaluated the activity of all these ERK IR mutants by qPCR analysis of the *Egr1* level in their transgenic ESCs. Among the three ERK1 IR mutants, all could significantly elevate the transcriptional levels of *Egr1* in the *Erk1*-IR transgenic ESCs upon the addition of serum (Figure 3B). On the other hand, the induction of *Egr1* in these transgenic ESCs was significantly repressed when PD was present in the medium. However, *Egr1* expression could still be induced in *Erk* DKO + *Erk1*-IR ESCs when Vx11e was added (Figure 3B), suggesting that these ERK1 mutants were genuine Vx11e-resistant mutants.

To better compare the three ERK1 IR mutants, their relative kinase activities under different conditions were quantified based on the *Egr1* levels. For each transgenic cell line, the kinase activity in the serum-starved group was used as a baseline, and that in the serum-alone group was defined as 100%. After quantification, it was revealed that the ERK1-G55A mutant had higher kinase activity under the serum + Vx11e condition than under the serum-alone condition (Figure 3C). Likewise, all three ERK2 IR mutants could significantly induce *Egr1* transcription in their transgenic ESCs when stimulated with serum, and the upregulation of *Egr1* levels could be inhibited by PD but not Vx11e (Figure 3D). Additionally, similar to ERK1-G55A mutant, ERK2-G35S mutant had higher kinase activity under the serum + Vx11e condition than under the serum-alone condition (Figure 3E). Based on the levels of relative kinase activity under the serum + Vx11e condition, the ERK1-G55A and ERK2-G35S mutants were the optimal IR mutants for ERK1 and ERK2, respectively.

To further verify the kinase activity and drug response of the two ERK mutants, we measured the phosphorylation levels of p90-ribosomal protein S6 kinase (RSK), a direct and common substrate of ERK1/2 (Goetz et al., 2014). The levels of phosphorylated RSK (pRSK) were significantly increased in the *Erk* DKO ESCs expressing the *Erk1* or *Erk2* transgene but not in the *Erk* DKO ESCs expressing RFP transgene when these cells were stimulated with bFGF (Supplementary Figure S2D). The supplementation of either PD or Vx11e reduced the pRSK levels to the basal level observed in serum-starved groups of the *Erk* DKO ESCs expressing the *Erk1* or *Erk2* transgene (Supplementary Figure S2D). These findings suggest that the changes in the levels of pRSK in transgenic ESCs can represent the relative kinase activity and inhibition response of ERK proteins.

Next, we used pRSK levels as a readout to evaluate the kinase activity and drug response of ERK1-G55A and ERK2-G35S mutants in ESCs. Both the ERK1-G55A transgenic ESCs and the ERK2-G35S transgenic ESCs had higher levels of pRSK when cells were stimulated with bFGF, suggesting that both ERK1-G55A and ERK2-G35S mutants retained their kinase activity in ESCs (Figure 3F). The addition of PD reduced the levels of pRSK in these two transgenic ESCs; however, the addition of Vx11e did not decrease the pRSK levels (Figure 3F). The increased pRSK levels in the *Erk*-IR (ERK1-G55A or ERK2-G35S) transgenic ESCs after the stimulation with Vx11e and bFGF suggested the activation of MAPK/ERK signaling in these cells even in the presence of Vx11e.

Finally, we examined ERK1-G55A and ERK2-G35S mutants in ESCs based on their phenotypes. *Erk* DKO ESCs provide a valuable tool to evaluate the kinase activity of ERK mutants based on the cell fate of ERK mutant transgenic ESCs under the CHIR alone condition. The RFP transgenic ESCs, without the expression of ERK1/2, similar to *Erk* DKO ESCs, retained self-renewal under the CHIR alone condition (Supplementary Figure S2E). The *Erk* DKO ESCs expressing WT *Erk1* or *Erk2* transgene differentiated in the presence of CHIR alone. Similar to the WT *Erk* transgenic ESCs, the *Erk*-IR transgenic ESCs differentiated in the CHIR alone medium (Figure 3G), suggesting that ERK1-G55A and ERK2-G35S mutants preserved their kinase activity in ESCs. Additionally, both the WT *Erk* and *Erk*-IR transgenic ESCs remained undifferentiated under the 2i condition. Likewise, the WT *Erk* transgenic ESCs remained undifferentiated under the Vx11e + CHIR condition (Supplementary Figure S2E). However, the *Erk* DKO ESCs expressing ERK1-G55A or ERK2-G35S differentiated under the same condition (Figure 3G). These data indicate that both ERK1-G55A and ERK2-G35S mutants retain their kinase activity and are resistant to ERK inhibition by Vx11e.

Collectively, these results suggest that ERK1-G55A and ERK2-G35S mutants in ESCs retain kinase activity, are resistant to Vx11e but responsive to PD03. We also conclude that these two ERK mutants can be used to establish ESC lines where individual ERK paralogs can be selectively inhibited by Vx11e.

### 2.6 Selective inhibition of either ERK1 or ERK2 is not sufficient to maintain ESC self-renewal in the presence of CHIR

**FIGURE 4:**
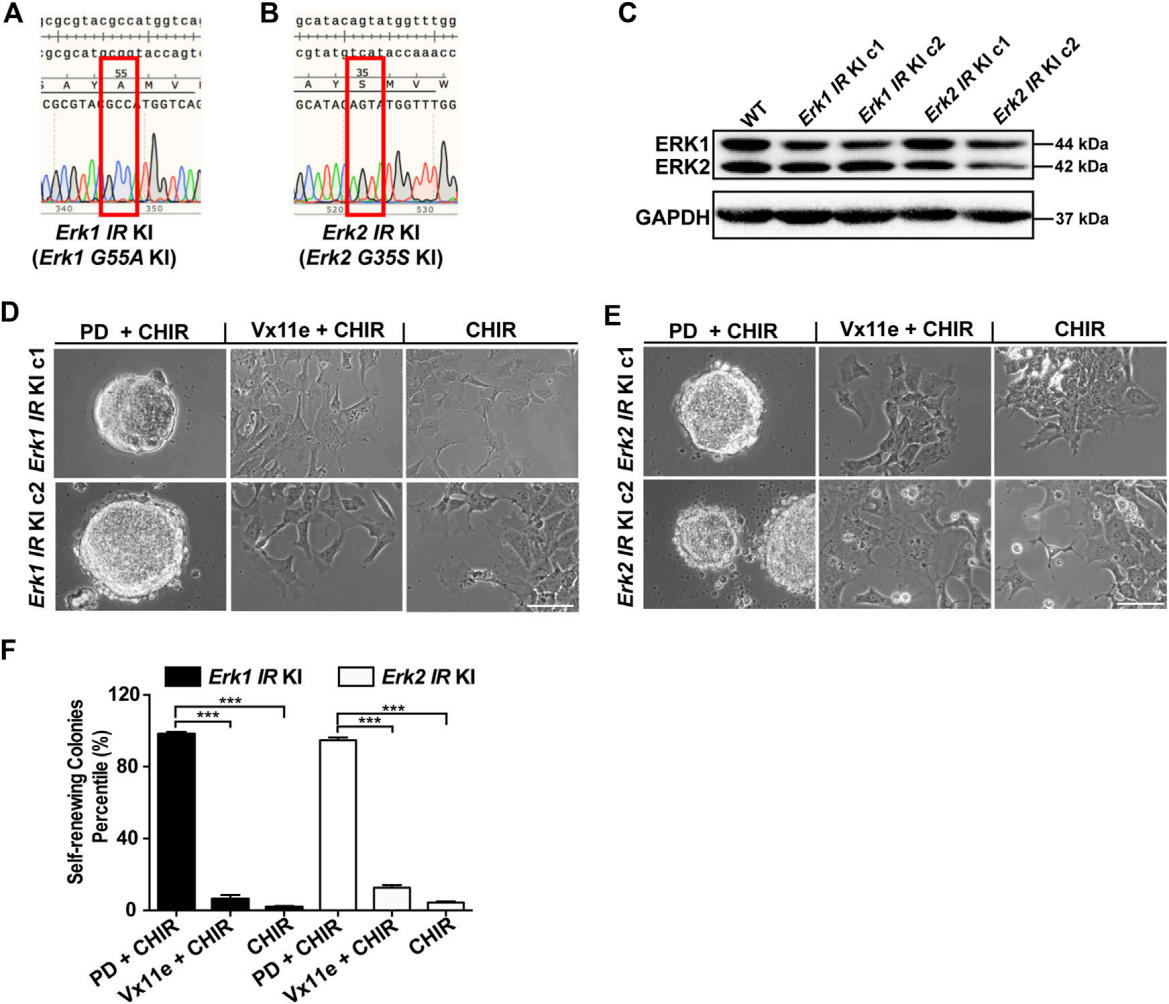
Selective inhibition of either ERK1 or ERK2 with the inhibitor-resistant approach is not sufficient to maintain ESC self-renewal in the presence of CHIR. **(A,B)** Representative DNA sequencing results on *Erk* genomic regions of *Erk1* ^*G55A*^ KI ESCs **(A)** and *Erk2* ^*G35S*^ KI ESCs **(B)**. For each type of *Erk IR* KI ESCs, we had two different clones, and the results of one clone of the 2 cell lines were shown. KI: knock-in. **(C)** Western blot analysis of the expression of ERK1/2 and GAPDH in the two different clones of *Erk IR* KI ESCs. **(D,E)** Representative phase contrast images of *Erk1* IR KI ESCs **(D)** and *Erk2* IR KI ESCs **(E)** under the indicated conditions in N2B27 medium for 7 days. Scale bars, 100 µm. **(F)** Quantification of the percentages of self-renewing colonies (AP+) formed in *Erk1* IR KI ESCs and *Erk2* IR KI ESCs cultured in N2B27 medium supplemented with PD + CHIR, Vx11e + CHIR, or CHIR. Data represent means ± SEM (n = 2). ***: *p* < 0.001.

Having demonstrated that individual ERK1/2 isoforms can be specifically inhibited by this new inhibitor-resistant strategy, we next investigated the resulting phenotypes regarding ESC self-renewal. To this end, we first generated *Erk1-G35S* knock-in (KI) ESCs and *Erk2-G35S* KI ESCs via CRISPR/Cas9-mediated homologous recombination. Five out of the 83 picked clones were homozygous (both alleles with *Erk1-G55A* mutation) in the pool of candidate *Erk1-G55A* KI ESCs, and six out of the 93 picked clones were homozygous in the pool of candidate *Erk2-G35S* KI ESCs. Two clones of both the *Erk1-G55A* KI ESCs (c1 and c2) and the *Erk2-G35S* KI ESCs (c1 and c2) were selected, and DNA sequencing on Erk genomic regions confirmed the homozygous intended mutations in them (Figures 4A, B). The expression of ERK1/2 in these *Erk*-IR KI ESCs was confirmed by immunoblotting. In both the two clones of *Erk1-G55A* KI ESCs and *Erk2-G35S* KI ESCs, the levels of ERK1/2 were similar to those in WT ESCs (Figure 4C). These results revealed the successful establishment of the *Erk1*-IR KI ESCs (*Erk1-G55A* KI ESCs) and *Erk2*-IR KI ESCs (*Erk2-G35S* KI ESCs).

Using these genetically engineered ESCs, we next examined the effect of selective inhibition of ERK1 or ERK2 on ESC self-renewal. In these *Erk*-IR KI ESCs, both ERK1/2 were inhibited under the 2i condition and were active under the CHIR alone condition. In the medium with Vx11e + CHIR, ERK1 and ERK2 were selectively inhibited in *Erk2*-IR KI ESCs and *Erk1*-IR KI ESCs, respectively. It was revealed that both *Erk1*-and *Erk2*-IR KI ESCs remained undifferentiated under the 2i condition and differentiated under the CHIR alone condition (Figures 4D, E). Additionally, the selective inhibition of ERK2 in *Erk1*-IR KI ESCs or the selective inhibition of ERK1 in *Erk2*-IR KI ESCs by Vx11e was not sufficient to maintain ESC self-renewal together with CHIR, leading to the formation of a mixed population of undifferentiated and differentiated colonies (Figures 4D, E). Consistent with the above results, in the AP staining assay, the percentages of self-renewing colonies in the *Erk1*-IR KI and *Erk2*-IR KI ESC populations cultured with the Vx11e + CHIR medium were significantly lower than those cultured with the 2i medium (Figure 4F). The data suggest that the individual inhibition of ERK1 or ERK2 is not sufficient to sustain ESC self-renewal in the presence of a GSK3 inhibitor.

To rule out possible off-target effects of CRISPR/Cas9 technology on ESC self-renewal, we further validated the conclusions regarding the individual roles of ERK1/2 isoforms in ESC self-renewal using an add-back strategy. To this end, we established *Erk1*-IR transgenic ESCs (*Erk1* ^*−/−*^ *+ Erk1-IR*) by transfecting *Erk1* ^*−/−*^ ESCs with the construct expressing the ERK1-IR mutant and RFP together. We also generated isogenic RFP transgenic ESCs (*Erk1* ^*−/−*^ *+ RFP*) and *Erk1-WT* transgenic ESCs (*Erk1* ^*−/−*^ *+ Erk1-WT*) as controls. Similarly, we made isogenic *Erk2-IR*, *Erk2-WT*, and *RFP* transgenic ESCs based on *Erk2* ^*−/−*^ ESCs with similar constructs. The expression of the transgenes (RFP, ERK1, or ERK2) in these different transgenic ESCs was confirmed with immunoblotting (and Supplementary Figures S3A, S3B).

We next determined the individual role of each ERK paralog in ESC self-renewal using these *Erk-IR* transgenic ESCs. *Erk1* ^*−/−*^ *+ RFP* ESCs and *Erk1* ^*−/−*^ *+ Erk1-WT* ESCs remained undifferentiated in the presence of either 2i or Vx11e + CHIR (Supplementary Figure S3C). Similarly, *Erk1* ^*−/−*^ *+ Erk1-IR* ESCs remained undifferentiated in 2i medium; however, they differentiated in the Vx11e + CHIR medium (Supplementary Figure S3C). In the AP staining assays for both *Erk1* ^*−/−*^ *+ RFP* ESCs and *Erk1* ^*−/−*^ *+ Erk1-WT* ESCs, the percentages of self-renewing colonies under the 2i condition were similar to those under the Vx11e + CHIR condition (Supplementary Figure S3D). In contrast, the *Erk1* ^*−/−*^ *+ Erk1-IR* ESCs had a significantly lower percentage of self-renewing colonies under the Vx11e + CHIR condition than under the 2i condition. Additionally, both *Erk2* ^*−/−*^ *+ RFP* ESCs and *Erk2* ^*−/−*^ *+ Erk2-WT* ESCs could be maintained in the Vx11e + CHIR medium (Supplementary Figure S3E), and their percentages of self-renewing colonies under the Vx11e + CHIR condition were comparable to those under the 2i condition (Supplementary Figure S3F). In contrast, the *Erk2* ^*−/−*^ *+ Erk2-IR* ESCs could not keep self-renewing under the Vx11e + CHIR condition (Supplementary Figure S3E), and the percentage of self-renewing colonies for them under the same condition was significantly lower than that under 2i condition.

Collectively, based on the findings from the *Erk-IR* KI ESCs and the *Erk-IR* transgenic ESCs, we conclude that individual chemical inhibition of either ERK1 or ERK2 is not sufficient to sustain ESC self-renewal under the CHIR alone condition.

### 2.7 Blocking ERK1 or ERK2 kinase function via a kinase-dead approach cannot sustain ESC self-renewal in the presence of CHIR

To further validate our conclusion about ERK1/2 from the novel inhibitor-resistant strategy, we intended to selectively eliminate kinase activity of one of the ERK1/2 paralogs with a kinase-dead (KD) approach. The KD mutant of a protein is generated by introducing a point mutation in the kinase domain so that its phosphoryl transfer potential is lost; however, this mutant can still act as a scaffold for binding substrates. To generate ERK1-KD and ERK2-KD mutants, mutagenesis was performed to introduce a K72R mutation and a K52R mutation into the coding sequences of wild-type *Erk1* and *Erk2*, respectively (Goetz et al., 2014).

We next utilized the KD approach to verify the role of ERK1/2 paralogs in ESC self-renewal. To specifically inhibit ERK1 or ERK2 in ESCs, we generated *Erk1* ^*−/−*^ *+ Erk1-KD* ESCs and *Erk2* ^*−/−*^ *+ Erk2-KD* ESCs by transfecting *Erk1* ^*−/−*^ ESCs and *Erk2* ^*−/−*^ ESCs with constructs expressing the ERK1-KD and the ERK2-KD mutants, respectively. The expression of the ERK-KD mutants in these transgenic ESCs was confirmed by immunoblotting (Supplementary Figure S4A). Following this, we characterized the cell fate of these ERK-KD transgenic ESCs cultured under the 2i and CHIR alone conditions. It was revealed that both the *Erk1* ^*−/−*^ *+ Erk1-KD* ESCs and the *Erk2* ^*−/−*^ *+ Erk2-KD* ESCs retained their self-renewal under the 2i condition; in contrast, both ESC lines differentiated under the CHIR alone condition (and Supplementary Figures S4B, S4C). In accordance with the above results, both the *Erk1* ^*−/−*^ *+ Erk1-KD* ESCs and the *Erk2* ^*−/−*^ *+ Erk2-KD* ESCs had significantly lower percentages of self-renewing colonies under the CHIR alone condition than under the 2i condition (Supplementary Figure S4D). These results suggest that inhibiting the kinase activity of one of the ERK1/2 isoforms is not sufficient to retain ESC self-renewal in the presence of GSK3 inhibitor.

### 2.8 Selective inhibition of either ERK1 or ERK2 alone cannot block ESC differentiation

The activation of MAPK/ERK signaling is critical for the differentiation of ESCs into cells of three germ layers (Lanner and Rossant, 2010). Previous studies have demonstrated that ERK1 and ERK2 in the zebrafish played non-redundant roles in gastrulation, a key process for three germ layer differentiation (Krens et al., 2008a; Krens et al., 2008b). We hypothesized that ERK1/2 may have different roles in ESC differentiation.

We first investigated the individual role of ERK1/2 isoforms in ESC differentiation using *Erk-IR* KI ESCs. Considering the difficulty of drug diffusion into the core of embryoid bodies and the large variation in the differentiation efficiency in embryoid body-based differentiation protocols (Brickman and Serup, 2017), we used monolayer differentiation protocols. For neural differentiation, an adherent monolayer neural differentiation protocol was employed (Ying et al., 2003). Since the parental cell line of the *Erk-IR* KI ESCs carries a *Sox1-GFP* knock-in reporter (Ying et al., 2003) and Sox1 is a lineage-specific marker of neuroectoderm in the mouse (Wood and Episkopou, 1999), we traced the neuroectoderm differentiation of *Erk-IR* KI ESCs by GFP expression. Results showed that both WT and the *Erk-IR* KI ESCs readily differentiated into Sox1-GFP + neural stem cells (NSCs) in the neural differentiation medium (Supplementary Figure S5A). Inhibition of the MAPK/ERK signaling by PD could not block the formation of Sox1-GFP + NSCs in both WT and the *Erk-IR* KI ESC populations when cultured in the neural differentiation medium (Supplementary Figure S5A). Consistent with this, Sox1-GFP + NSCs could be generated from WT ESCs when ERK1/2 were inhibited by Vx11e in these cells. Additionally, both the *Erk1-IR* KI ESCs and the *Erk2-IR* KI ESCs could still produce abundant Sox1-GFP + NSCs in the neural differentiation medium when one of the ERK1/2 isoforms in the *Erk-IR* ESCs was inhibited with Vx11e (Supplementary Figure S5A). These results suggest that inhibition of both ERK1/2 or selective inhibition of one of the ERK1/2 isoforms could not block neural differentiation of ESCs.

We next investigated the roles of ERK1/2 during mesendoderm differentiation. In mesoderm differentiation medium, both WT ESCs and the *Erk-IR* KI ESCs produced considerable mesoderm progenitors (Brachyury+), and the numbers of mesoderm progenitors formed in these ESCs dramatically decreased when MAPK/ERK signaling was inhibited by PD (Supplementary Figure S5B). In line with the above results, direct inhibition of ERK1/2 by Vx11e in the WT ESCs suppressed their differentiation into Brachyury + mesoderm progenitors. In contrast, for the *Erk-IR* KI ESCs, the supplementation of Vx11e in the mesoderm differentiation medium did not block their differentiation into Brachyury + cells (Supplementary Figure S5B). These results indicate that specific inhibition of one of the ERK1/2 isoforms in ESCs cannot block mesoderm differentiation.

As for endoderm differentiation, both WT ESCs and the *Erk-IR* ESCs could differentiate and form abundant endoderm progenitors (FoxA2+) in the endoderm differentiation medium (Supplementary Figure S5C), and the formation of endoderm progenitors was abolished in these ESCs when PD was supplemented to the differentiation medium. Similarly, FoxA2+ endoderm cells could not be generated from the WT ESCs in the endoderm differentiation medium supplemented with Vx11e. However, under the same condition, we found many FoxA2+ endoderm progenitors emerged from the populations of the *Erk1-IR* KI ESCs and the *Erk2-IR* KI ESCs (Supplementary Figure S5C). These findings imply that the activation of either ERK1 or ERK2 in ESCs was sufficient to induce endoderm differentiation. To further confirm the individual roles of ERK1 and ERK2 in three germ layer differentiation, we repeated the differentiation assays using the *Erk1* ^*−/−*^ *+ Erk1-KD* ESCs and the *Erk2* ^*−/−*^ *+ Erk2-KD* ESCs, in which one of the ERK1/2 isoforms was inhibited. Both types of these transgenic ESCs could differentiate into progenitor cells in the three germ layers based on immunostaining of different lineage-specific markers (Supplementary Figures S5D, S5F). Taken together, our findings demonstrate that selective inhibition of one of the ERK1/2 isoforms in ESCs cannot block their differentiation into cells of the three germ layers.

### 2.9 Selective inhibition of ERK1, but not ERK2, promotes mesendoderm differentiation of ESCs

**FIGURE 5:**
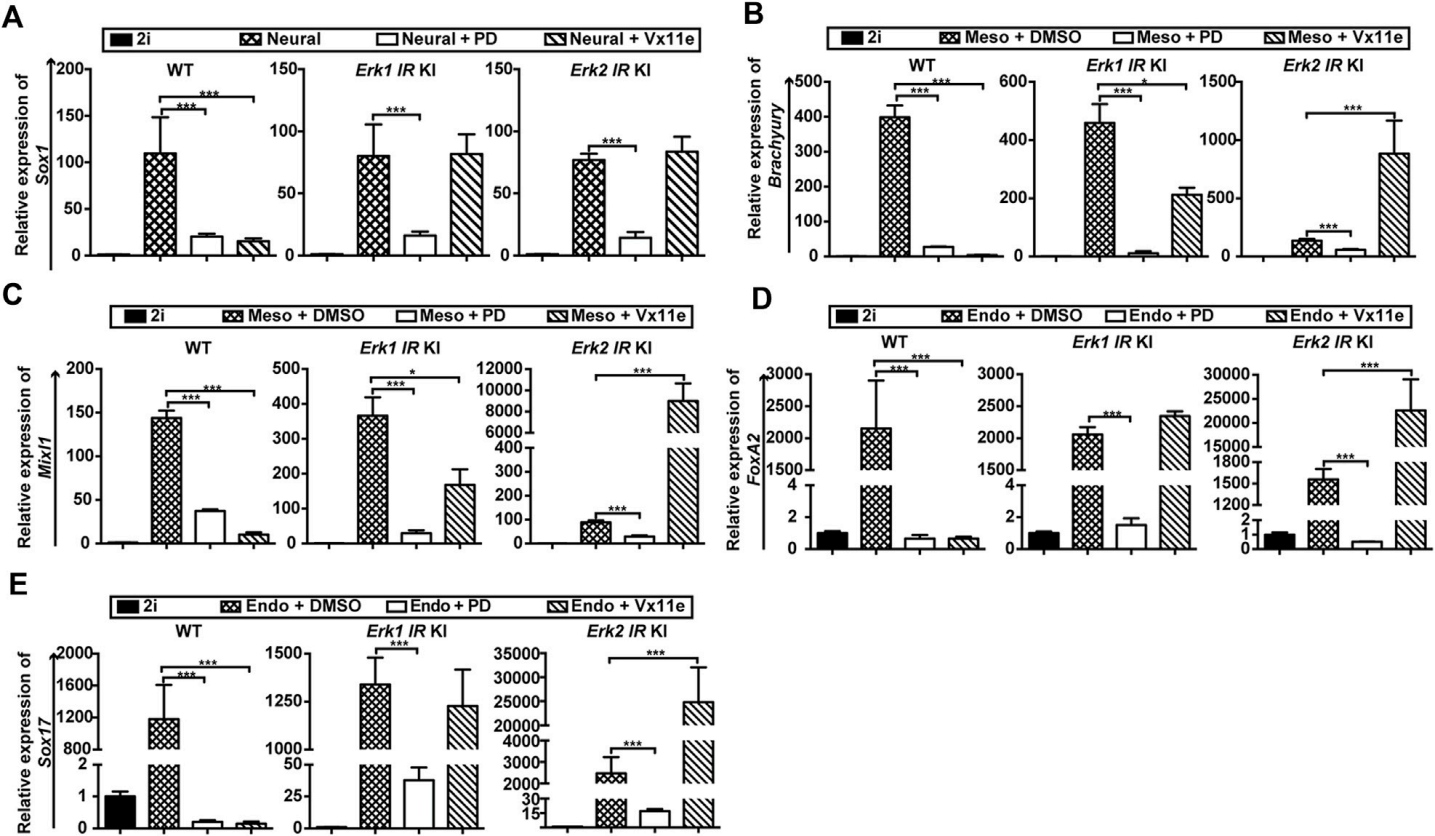
Selective inhibition of ERK1, not ERK2, promotes ESC differentiation into mesendodermal cells. **(A)** qPCR analysis of a neuroectoderm marker (*Sox1*) in WT ESCs, the *Erk1* IR KI ESCs c1, and the *Erk2* IR KI ESCs c1 cultured in neural differentiation medium (N2B27 medium) under the indicated conditions for 4 days. The *Erk1 IR* KI ESCs c2 and the *Erk2 IR* KI ESCs c2 had similar results. Data are shown as means ± SEM (n = 2). ***: *p* < 0.001 **(B,C)** qPCR analysis of mesoderm markers, *Brachyury* **(B)** and *Mixl1* **(C)**, in WT ESCs, *Erk1* IR KI ESCs c1, and *Erk2* IR KI ESCs c1 cultured in mesoderm differentiation medium under the indicated conditions for 5 days. The *Erk1* IR KI ESCs c2 and the *Erk2* IR KI ESCs c2 showed similar results. The ESCs cultured with 2i were used as the controls. Data are shown as means ± SEM (n = 3). *: *p* < 0.05; ***: *p* < 0.001. **(D,E)** qPCR analysis of endoderm markers, *FoxA2* **(D)** and *Sox17* **(E)**, in WT ESCs, *Erk1* IR KI ESCs c1, and *Erk2 IR* KI ESCs c1 cultured in endoderm differentiation medium under the indicated conditions for 5 days. The *Erk1* IR KI ESCs c2 and the *Erk2* IR KI ESCs c2 showed similar results. The ESCs cultured with 2i were used as the controls. Data are shown as means ± SEM (n = 3). ***: *p* < 0.001.

To better determine the roles of ERK1/2 in three germ layer differentiation, we quantified the expression of lineage-specific markers in three types of ESCs under different differentiation conditions by qPCR. Regarding neural differentiation, the inhibition of MAPK/ERK signaling in WT ESCs (by PD or Vx11e) and in *Erk-IR* KI ESCs (by PD) significantly reduced Sox1 transcription (Figure 5A), underscoring the importance of MAPK/ERK signaling activation during neural differentiation. Additionally, the addition of PD could not completely inhibit the upregulation of Sox1 in the three types of ESCs, consistent with the emergence of Sox1-GFP + cells when the MAPK/ERK pathway was inhibited in these ESCs (Supplementary Figure S5A). Moreover, the selective inhibition of either ERK1 or ERK2 with Vx11e in *Erk-IR* KI ESCs did not significantly alter Sox1 levels during neural differentiation (Figure 5A), suggesting that selective inhibition of either ERK1 or ERK2 in ESCs had trivial effects on the formation of Sox1+ NSCs.

Regarding mesendoderm differentiation, the inhibition of the MAPK/ERK pathway in WT ESCs (by PD or Vx11e) and in *Erk-IR* ESCs (by PD) significantly inhibited the induction of mesoderm markers (*Brachyury*, *Mixl1*) (Figures 5B, C) and endoderm markers (*FoxA2*, and *Sox17*) (Figures 5D, E). Interestingly, the selective inhibition of ERK2 in *Erk1-IR* KI ESCs by Vx11e decreased the levels of *Brachyury* and *Mixl1* (Figures 5B, C) but had minor effects on the transcription of *FoxA2* and *Sox17* (Figures 5D, E). In contrast, selective inhibition of ERK1 in *Erk2-IR* KI ESCs with Vx11e significantly increased the levels of mesoderm and endoderm markers (Figures 5B–E). These results suggest that individual inhibition of ERK1 but not ERK2 in ESCs promotes mesendoderm differentiation.

Based on the above findings, we can conclude that ERK1/2 in ESCs are functionally redundant in neural differentiation but are non-redundant in mesendoderm differentiation.

## 3 Discussion

In this study, through a combination of genetic and chemical-genetic approaches in ESCs, we demonstrated that ERK1/2 are dispensable for the survival and expansion of mouse ESCs. Moreover, we showed that inhibition of ERK1/2 is sufficient to mimic the effect of MEK inhibitor in maintaining ESC self-renewal when GSK3 is inhibited by CHIR. Additionally, we introduced a novel inhibitor-resistant (IR) approach to achieve selective inhibition of individual ERK paralogs for the first time. Our results indicate that the two ERK isoforms play both redundant and non-redundant roles in regulating ESC fate. Genetic deletion or chemical inhibition of either ERK isoform alone is inadequate to sustain ESC self-renewal or block neural differentiation. However, for mesendoderm differentiation of ESCs, selective inhibition of ERK1 exhibits different effects compared to selective inhibition of ERK2. Thus, our study offers novel insights into the roles of ERK isoforms in controlling cell fate.

A previous study suggested that ERK1/2 are indispensable for genomic stability and self-renewal of mouse ESCs (Chen et al., 2015). However, our findings contradict this claim. We demonstrated that deletion of both ERK isoforms in mouse ESCs does not affect their survival and self-renewal ability. Furthermore, mouse ESCs could be maintained by Vx11e plus CHIR, supporting the notion that ERK1/2-dependent signaling is dispensable for mouse ESC self-renewal. Additionally, we showed that ERK1/2 are dispensable for the genomic stability of mouse ESCs. Discrepancies between the two studies may be attributed to differences in the genetic backgrounds of the mouse ESCs used. Notably, mice with mutations related to MAPK/ERK signaling exhibit varied phenotypes between C57BL6/J and 129/SvEv strains (Araki et al., 2009; Wu et al., 2011; Hernandez-Porras et al., 2015; Castel et al., 2019).

The development of the IR strategy enables deciphering the individual roles of highly homologous kinases and offers several advantages over the bump-hole method. IR mutants are screened based on phenotype, likely preserving kinase activity and substrate specificity. In contrast, the bump-hole approach necessitates a mutation at the gatekeeper position, potentially compromising kinase catalytic activity. Furthermore, the IR strategy minimizes off-target effects associated with bump inhibitors, eliminating the need for an additional inhibitor. Combining the IR strategy with traditional chemical genetic approaches facilitates the individual inhibition of highly homologous kinase isoforms in a single cell type, circumventing the need to establish separate cell lines. Although the IR approach requires more time and expenses for screening and identifying optimal mutants, insights from structural biology studies on inhibitor mechanisms aid in designing IR mutants, potentially shortening screening time.

Our findings regarding the redundant and non-redundant roles of ERK1 and ERK2 in ESC self-renewal and lineage differentiation are likely to have broad implications. Manipulating the MAPK/ERK pathway *via* selective ERK inhibition alters lineage commitment efficiency without altering cell fate, particularly in the formation of mesendodermal cells. These holds promise for enhancing cell differentiation efficiency in regenerative medicine. Additionally, the distinct roles of ERK isoforms highlight the complexity of the MAPK/ERK pathway, with implications for various biological processes and diseases. Exploring the specific roles of each ERK isoform in different cell types and diseases using ESCs or patient-derived iPSCs may pave the way for developing isoform-specific inhibitors for disease treatment.

It is important to note that mouse and human ESCs respond differentially to ERK inhibition. For example, while MEK/ERK inhibition promotes self-renewal in mouse ESCs, it can induce differentiation in human ESCs (Nichols and Smith, 2009; Hanna et al., 2010; Lanner and Rossant, 2010). Consequently, the conclusions drawn from our study in mouse ESCs may not be directly applicable to human ESCs. Future research should investigate the roles of ERK1 and ERK2 in human ESCs using similar genetic and chemical-genetic approaches. This could involve developing human ESC lines with selective inhibition of ERK paralogs to delineate their specific functions in self-renewal and differentiation. Additionally, comparative studies between mouse and human ESCs can provide valuable insights into the conserved and divergent aspects of ERK signaling in pluripotency and lineage commitment.

While our study has elucidated the distinct and overlapping roles of ERK1 and ERK2 in ESC self-renewal and differentiation, further research is necessary to identify and characterize the downstream effectors of these kinases. Understanding the specific targets and pathways downstream of ERK1 and ERK2 will provide deeper insights into their mechanistic roles in cell fate decisions. Future investigations should employ advanced proteomic and genomic approaches to systematically map the downstream signaling networks. These studies will not only enhance our understanding of ERK1/2-mediated signaling in ESCs but also reveal potential therapeutic targets for manipulating cell fate in regenerative medicine and disease contexts.

## 4 Methods

### 4.1 Cell culture

46C ESCs were maintained under feeder-free conditions and cultured on 0.1% gelatin-coated plates at 37°C in a 5% CO2 incubator, as described previously (Ying et al., 2003). Either serum medium (10%) with 10 ng/mL leukemia inhibitory factor (LIF) or serum-free N2B27 medium supplemented with CHIR99021 (3 μM) and PD0325901 (1 μM) was used.

### 4.2 EB formation and differentiation

For embryoid body (EB) formation, (5–10) ×10^5^ ESCs were plated in one 35 × 10 mm Petri-dish (Genesee Scientific) and cultured in the serum medium without LIF or inhibitors. The aggregates were allowed to grow for 8 days in suspension, and samples were collected on day 4 and day 8 for quantitative real-time PCR analysis. In further differentiation assays, 4-5 EB aggregates were transferred to each well of laminin-coated NuncTM 4-well dishes (ThermoFisher Scientific) for neuroectoderm differentiation in N2B27 medium, and to each well of gelatin coated NuncTM 4-well dishes for mesendoderm differentiation in basal medium containing serum.

### 4.3 *In vitro* monolayer differentiation of ESCs

Differentiation was performed as described (Ying et al., 2003; Li et al., 2017) with minor modifications. Briefly, 46C ESCs were plated at 2 × 10^3^/cm^2^ in basal medium with LIF on laminin-coated plates (1:100) for neuronal differentiation. After 24 h, cells were cultured with N2B27 medium and kept for 4 days for qPCR analysis and 6 days for immunostaining. For mesoderm differentiation, cells were plated at 2 × 10^3^/cm^2^ in basal medium with LIF on 0.1% gelatin-coated plates. After 24 h, cells were cultured with N2B27 medium containing 20 ng/mL Activin A (Peprotech), 5 ng/mL bFGF (Peprotech) and 1 μg/mL Heparin (Sigma). On day 4, 3 μM CHIR was added into the differentiation medium together with other small molecules. The cells were kept for 5 days for qPCR analysis and 5 days for immunostaining. For definitive endoderm differentiation, cells were plated at 5 × 10^3^/cm^2^ in basal medium with LIF on 0.1% gelatin-coated plates. For the first 2 days, N2B27 based medium containing 20 ng/mL Activin A, 3 µM CHIR, 10 ng/mL FGF4 (Peprotech), 1 μg/mL Heparin, 100 nM PI103 (Cayman Chemical) was used. On day 3, cells were switched to SF5 based medium containing 20 ng/mL Activin A, 3 µM CHIR, 10 ng/mL FGF4, 1 μg/mL Heparin, 100 nM PI103 and 20 ng/mL EGF (Peprotech). 100 mL of SF5 basal medium was made by mixing DMEM/F12 medium (ThermoFisher Scientific) with 500 µL N2 supplement (100X) (ThermoFisher Scientific), 1 mL B27 without Vitamin A supplement (50X) (ThermoFisher Scientific), 1 g BSA (final concentration 1%) (CST), and 100 µL β-mercaptoethanol.

### 4.4 Generation of ERK1/2 mutant mouse ESC lines

To establish ERK1/2-expressing stable cell lines, the coding sequence (CDS) of Erk1 and Erk2 was amplified from ESCs cDNA by PCR with PrimeSTAR GXL DNA Polymerase (Clontech) and inserted into the PaggyBac (PB) vector. The PB transposon-based plasmids were purchased from SBI (PB Transposon vector # PB511B-1, PB Transposase vector #PB200PA-1), and the CMV-MCS-EF1a-pac dual promoter cassette in the PB Transposon vector was replaced by CAG-MCS-IRES-bsd or CAG-MCS-IRES-BleoR using *Nhe*I and *Cla*I, ensuring the high expression level of transgenes. Additionally, a turboRFP-P2A sequence was inserted into pPB-CAG-MCS-IRES-bsd/BleoR backbone to trace the expression of transgenes. A one step mutagenesis protocol was used for the generation of Erk mutants, including Erk1 G55A (inhibitor resistant, IR), Erk2 G35S (Erk2 IR), Erk1 Y54H, Erk2 Y34N, Erk1 P76L, Erk2 P56L, Erk1-K72R (kinase dead, KD) and Erk2- K52R (Erk2 KD). Erk1 mutants were inserted into pPB-CAG-turboRFP-P2A-MCS-IRES-BleoR using *Sbf*I and *Asc*I; Erk2 mutants were cloned into pPB-CAG-turboRFP-P2A-MCS-IRES-bsd with *Sbf*I and *Asc*I. 46C ESCs were transfected with PB vectors carrying Erk1/2 mutants using Lipofectamine LTX following the protocol provided by the manufacturer. After 24 h, zeocin (75 μg/mL, ThermoFisher Scientific) or Blasticidin (10 μg/mL, ThermoFisher Scientific) was added to fresh medium in order to start selection. After 5–7 days, the drug resistant cells were split and re-plated at 1,000/well in 6-well plates. After expansion, the potential clones were picked under fluorescence microscope and confirmed with Western blot.

### 4.5 Genome editing in mouse ESCs

To knock out genes in ESCs, we used CRISPR/Cas9 technology with the pX330 vector (plasmid # 42230; Addgene, Cambridge, MA), co-expressing Cas9 protein and guide RNA (gRNA). Additionally, a P2A-*pac* cassette expressing puromycin was inserted in-frame with Cas9 into the PX330 vector to help screen positive clones in transfected cell pools.

Two different gRNAs specifically targeting exon two of Erk1 and exon three of Erk2, respectively, were designed using an online gRNA-designing tool (crispr.mit.edu). The sequences of these two gRNAs are listed in Supplementary Table S1. Plasmids were confirmed by DNA sequencing and then transfected into ESCs using lipofectamine. After drug selection, colony picking, and cell expansion, ERK knock-out ESCs were screened by immunoblotting followed by confirmation with DNA sequencing.

To generate *Erk1-G55A* KI ESC lines and *Erk2-G35S* KI cell lines, both gRNAs and donor templates were used. The gRNAs were designed using the online software CRISPOR (crispor.tefor.net). The gRNA sequences used to generate *Erk1-G55A* KI and *Erk2-G35S* KI cell lines are listed in Supplementary Table S1. Additionally, template donors for introducing the ERK1 G55A mutation and the ERK2 G35S mutation were synthesized as single-stranded DNA (ssODN). The sequences of the two donors are listed in Supplementary Table S1.

### 4.6 Alkaline phosphatase staining

ESCs were plated at a density of 700–1,000 cells/well in 12-well plates coated with 0.1% gelatin and cultured with N2B27 medium supplemented with inhibitors. The inhibitors used in this study included PD, CHIR, and Vx11e (APExBIO, Houston, TX), and the conditions tested were PD + CHIR, Vx11e + CHIR, and CHIR. For each tested condition, cells from different ESC lines were plated in triplicate. The medium was changed every 2 days. After 6 days of culture, cells were washed with 1x PBS solution and stained for alkaline phosphatase using an Alkaline Phosphatase Detection Kit (Sigma, St. Louis, MO) according to the manufacturer’s instructions. At least two independent self-renewal assays were performed.

### 4.7 Western blot

Cells were washed with ice-cold 1x PBS solution twice and then lysed in pre-cold RIPA buffer (Teknova) supplemented with protease and phosphatase inhibitors Complete and phosSTOP (Roche). Protein concentrations were quantified with Piece BCA Protein Assay Kit (ThermoFisher Scientific). 15–50 μg total protein was loaded into each lane, separated using 10% SDS-PAGE gel, and then transferred onto PVDF membrane (BioRad). Probing was performed with specific primary antibodies and HRP-conjugated secondary antibodies. Primary antibodies used in the study were listed in the Supplementary Table S2.

### 4.8 Quantitative real-time PCR (qRT-PCR)

Total RNA was extracted from cells using Quick-RNA MiniPrep Kit (Zymo Research), and cDNA was transcribed from 0.5 to 1 μg RNA using iScript cDNA Synthesis Kit (BioRad) following the manufacturers’ instructions. Relative expression levels were determined using iTaq universal SYBR Green Supermix (Biorad), and expression levels were normalized to GAPDH. qRT-PCR experiments were performed in triplicate on a ViiA 7 Real-Time PCR System (ThermoFisher Scientific). All primers of targets used in this study are listed in the Supplementary Table S3.

### 4.9 Immunostaining

Cell cultures were fixed in 4% (vol/vol) paraformaldehyde for 15 min at room temperature (RT). Cells were blocked and permeabilized using 5% bovine serum albumin with 0.2% Triton X-100 in PBS for 1 h at 37°C. After washing, cells were incubated overnight at 4°C with primary antibodies (see Supplementary Table S2). Next, Alexa Fluor secondary antibodies (Thermo Fisher Scientific) were used at a 1:1,000 dilution in PBS for 1 hour at RT, followed by 3× washes with PBST and a 5 min incubation at RT with a 1:10,000 dilution of Hoechst (Thermo Fisher Scientific) in PBS.

### 4.10 Statistical analysis

Images were taken as results of a representative experiment. Data are based on at least two independent experiments (n ≥ 2). Statistical differences were analyzed using two-tailed Student’s t-tests for comparisons of the two groups (e.g., PD + CHIR vs. CHIR, Vx11e + CHIR vs. CHIR, serum vs. non-treatment, (serum + PD) vs. non-treatment, or (serum + Vx11e) vs. non-treatment) in each experiment. All data are shown as mean ± SEM. Data with *p* < 0.05 was considered statistically significant. *: *p* < 0.05, **: *p* < 0.01, ***: *p* < 0.001.

## Data Availability

The original contributions presented in the study are included in the article/Supplementary Material, further inquiries can be directed to the corresponding author.
