# Supplementary material for: A novel chemical genetic approach reveals paralog-specific role of ERK1/2 in mouse embryonic stem cell fate control
Source: Front Cell Dev Biol. 2024 Jul 12;12:1415621. doi: 10.3389/fcell.2024.1415621 (PMC11272557; doi:10.3389/fcell.2024.1415621)
Supplement: Supplementary file 1 [file DataSheet1.pdf]

# Supplementary Material

## A novel chemical genetic approach reveals paralog-specific role of ERK1/2 in mouse embryonic stem cell fate control

**Liang Hu[1,3], Xiong Xiao[1], Wesley Huang[1], Tao Zhou[1], Weilu Chen[1], Chao Zhang[2], Qi-Long Ying[1,*]**

*** Correspondence:**

Qi-Long Ying

qying@med.usc.edu

**1 Supplementary Tables**

**2 Supplementary Figures**

## Table S1 DNA oligonucleotides for genome editing with CRISPR/Cas9 technique

| Name | Sequences (5'-3') |
|---|---|
| *Erk1* gRNA for knock-out | TATATACTTGAGGCCCCGG |
| *Erk2* gRNA for knock-out | TTTGCTCAATGGTTGGTGCC |
| *Erk1* knock-out genotyping-F | TCCTTTTGAGCACCAGACCT |
| *Erk1* knock-out genotyping-R | AGCGCAAACATTATAATCTCCTCT |
| *Erk2* knock-out genotyping-F | TCAAGTCTCAGTGTAGGCCC |
| *Erk2* knock-out genotyping-R | CACTGTCACTGTGAGCCTTGT |
| *Erk1 G55A* KI gRNA | TACATCGGCGAGGGCGCGTA |
| *Erk2 G35S* KI gRNA | TACTCACCAAACCATGCCGT |
| *Erk1 G55A* KI ssODN | ctccctgagccccccatgtcccgagcacacacctgctgtccctgctccgaagccccctcccgg gacgccccctcacctgaccat ggcgtacgcgccctcgccgatgtactgcagctgcgtgtagcgt |
| *Erk2 G35S* KI ssODN | gcggcggcgggcccggagatggtccgcgggcaggtgttcgacgtagggccgcgctacaccaa cctctcgtacatcggagaaggcgcatacagtatggtttggtgagtatccgcgctggatttcaggc |
| *Erk1 G55A* KI genotyping-F | GGCCTAAAGGCAGGAGGATG |
| *Erk1 G55A* KI genotyping-R | GCAGCGAGAACTCACAAACC |
| *Erk2 G35S* KI genotyping-F | TGTGGGGTCCTTATGCCTAAAT |
| *Erk2 G35S* KI genotyping-R | CAGTGTACTTCCGTTCCCGT |

## Table S2 Antibodies and their dilution used in this study

| Antibodies | Suppliers | Catalog No. | Dilution | Antibodies | Suppliers | Catalog No. | Dilution |
|---|---|---|---|---|---|---|---|
| ERK1/2 | CST | 9102S | 1:1000 | OCT4 | Santa Cruz | sc-5279 | 1:200 |
| pERK1/2 | CST | 4370 | 1:1000 | NANOG | R&D | AF2729-SP | 1:1000 |
| GAPDH | CST | 5174 | 1:2000 | β3-tubulin | CST | 4466S | 1:200 |
| RSK1/2/3 | CST | 9355T | 1:1000 | Myosin | DSHB | MF-20 | 1:50 |
| p-p90RSK (Ser380) | CST | 9241 | 1:300 | Brachyury | Abcam | Ab209665 | 1:1000 |
| REX1 | Santa Cruz | Sc-50669 | 1:100 | FoxA2 | CST | 8196T | 1:1000 |

## Table S3 The sequences of primers used in this study

| Target | Forward Sequence (5'-3') | Reverse Sequence (5'-3') |
|---|---|---|
| *GAPDH* | TGAAGCAGGCA TCTGAGGG | CGAAGGTGGAAGAGTGGGAG |
| *Rex1* | TCACTGTGCTGCCTCCAAGT | GGGCACTGA TCCGCAAAC |

| | | |
|---|---|---|
| *Oct4* | GAAGCAGAAGAGGA TCACCTTG | TTCTTAAGGCTGAGCTGCAAG |
| *Nanog* | CGGCTCACTTCCTTCTGACT | GGCGAGGAGAGGCAGC |
| *Egr1* | CCACAACAACAGGGAGACCT | ACTGAGTGGCGAAGGCTTTA |
| *Sox1* | CTCCTCGGCTGAATTCTTTG | TGTAATCCGGGTGTTCCTTC |
| *Brachyury* | CCGGTGCTGAAGGTAAATGT | CCTCCATTGAGCTTGTTGGT |
| *Mixl1* | TTGAATTGAACCCTGTTGTCCC | GAAACCCGTTCTCCCATCCACC |
| *FoxA2* | CCTCAAGGGAGCAGTCTCAC | TTTCTCCTGGTCCGGTACAC |
| *Sox17* | AGCCATTTCCTCCGTGGTGT | AACACTGCTTCTGGCCCTCAG |

**Supplementary Figures**

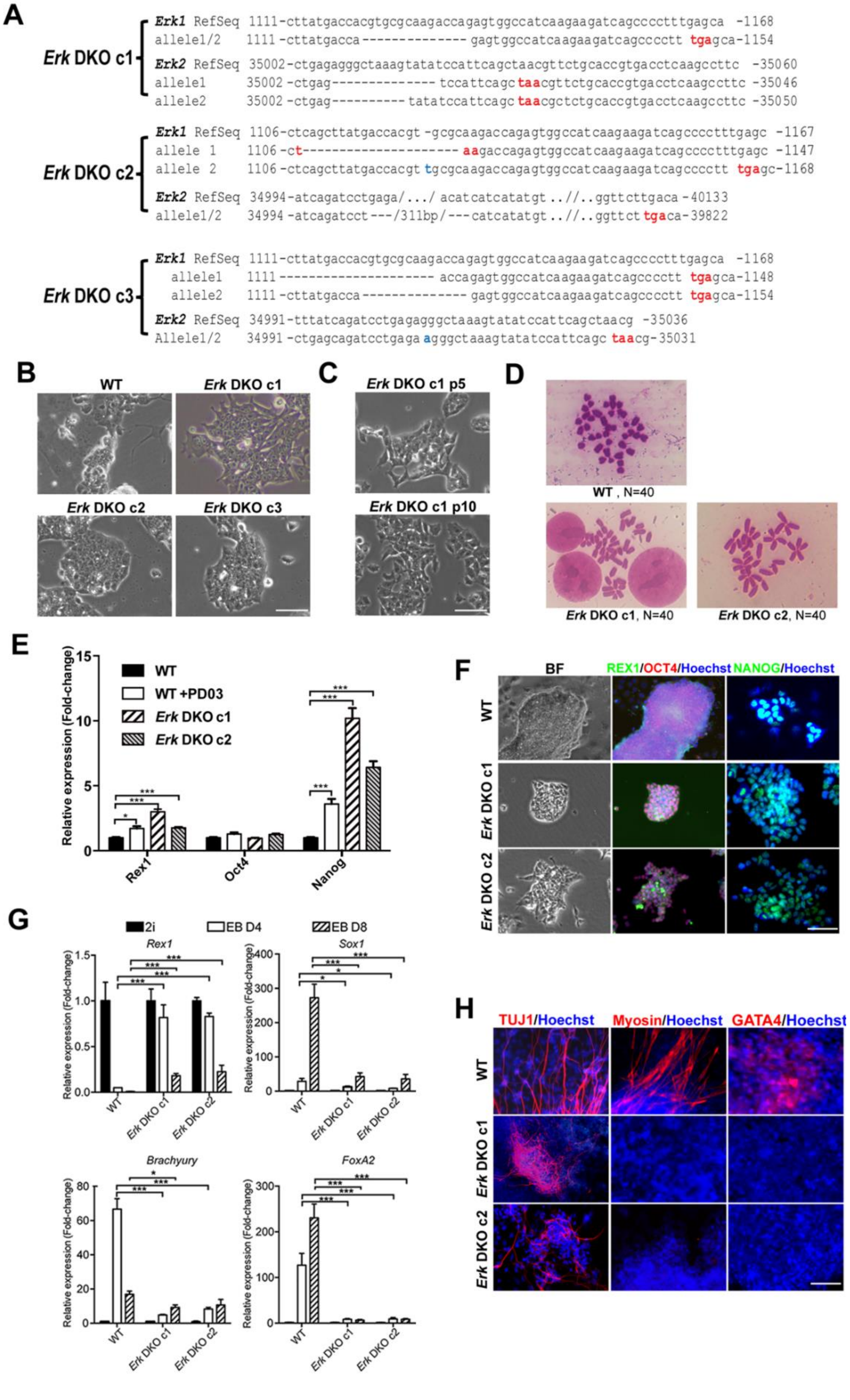

**Figure S1. Characterization of ESC lines with deletion of ERK1/2.**

(A) Sanger sequencing of genotyping PCR products covering the guide RNAs targeted loci in genomic regions of Erk1/2. The sequencing results for different clones of *Erk* double knockout (DKO) ESC lines indicated the disrupted Erk1/2 genomic loci and introduced frame-shift mutations, which leads to the insertion of premature stop codons in the mRNA transcript and knock-out of both Erk isoforms. The deletion of bases is shown in "-", and insertion of bases is denoted in light blue. The premature stop codon is labeled in red.

(B) Representative phase contrast images of wild-type (WT) mouse ESCs and three different clones of *Erk* DKO ESC lines ($2^{nd}$ passage, p2) cultured under leukemia inhibitory factor (LIF, 10 ng/ml) plus serum condition. Scale bar, 100μm.

(C) Representative phase contrast images of *Erk* DKO ESCs cultured in LIF + serum medium at p5 and p10. Scale bar, 100μm.

(D) Representative images of karyotyping results of WT ESCs and two clones of *Erk* DKO ESC lines cultured under LIF + serum condition (p12). Scale bar, 200μm.

(E) qPCR analysis of the expression of pluripotency markers (*Rex1*, *Oct4*, and *Nanog*) in different mouse ESC lines under LIF + serum condition. WT ESCs cultured in serum medium supplemented with LIF and PD03 were used as the control. Data represents mean ± SEM of three independent experiments (n=3). *: p< 0.5; ***: p<0.001.

(F) Representative phase contrast and immunofluorescent staining images of WT ESCs and two clones of Erk DKO ES cell lines maintained in LIF plus serum medium. Scale bar, 100μm.

(G) qPCR analysis of the expressions of *Rex1* and lineage markers (*Sox1* for neuroectoderm, *Brachyury* for mesoderm, *FoxA2* for definitive endoderm) in embryoid bodies of indicated cell lines collected on day 4 and day 8. The isogenic ESCs maintained under 2i condition were used as the controls. Data represents mean ± SEM of three independent experiments (n=3). *: p< 0.5; ***: p<0.001.

(H) Representative immunofluorescent staining images of the differentiated cells after plating embryoid bodies formed from WT and Erk DKO ESCs in three germ layer differentiation media. For neuroectoderm differentiation, TUJ1 (expressed in neuronal lineage cells) was used for staining after five days in N2B27 medium; for mesendoderm differentiation, Myosin (expressed in cardiomyocytes) and GATA4 (expressed in endoderm progenitors) were used for staining after ten days in serum medium. Scale bar, 100μm.

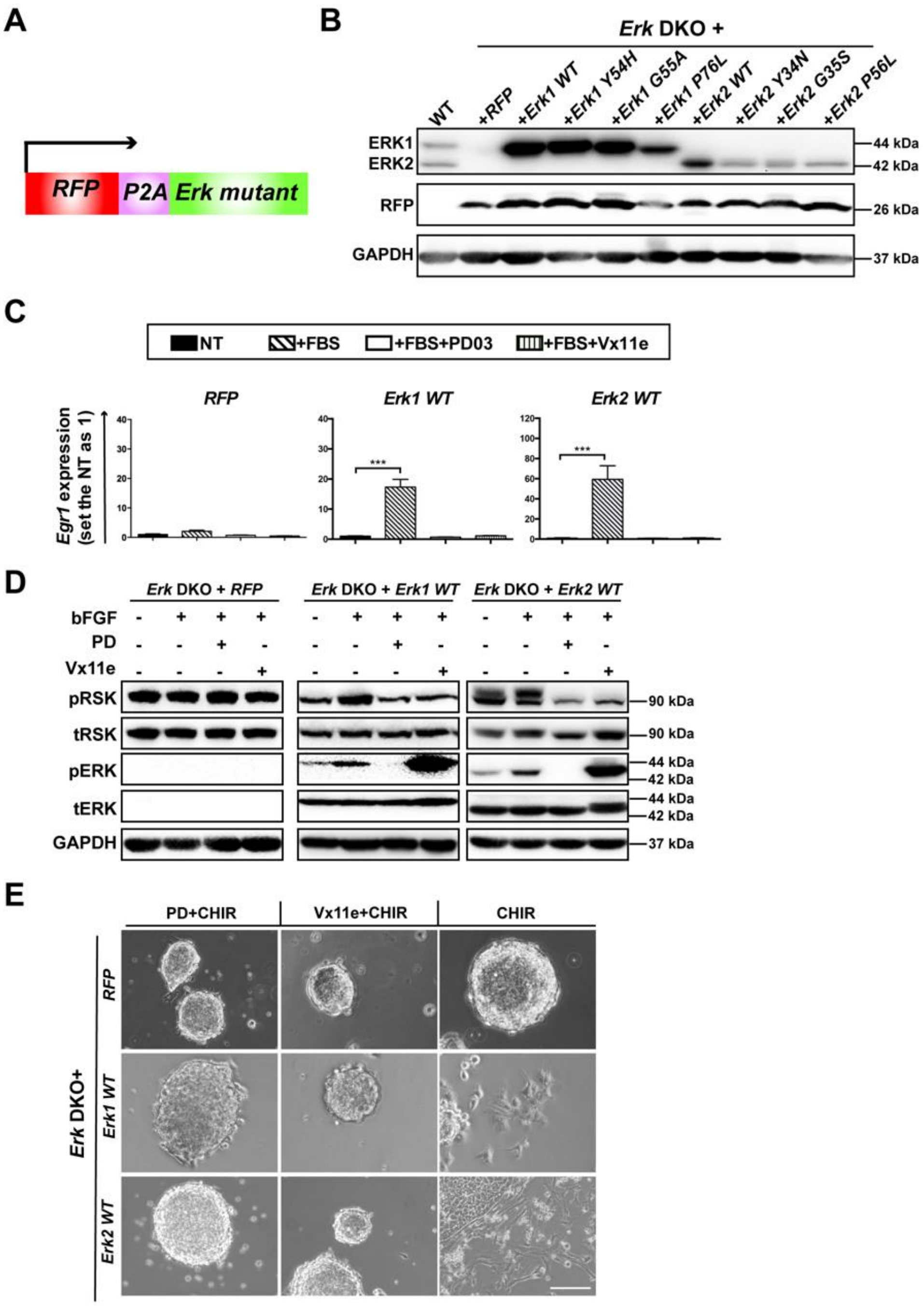


**Figure S2. A system used for screening optimal ERK1 and ERK2 inhibitor-resistant mutants for inhibitor-resistant strategy.**

(A) Illustration of the constructs for establishing *Erk* transgenic ESC lines. RFP: red fluorescence protein.

(B) Western blot analysis of the expression of ERK1/2, red-fluorescent protein (RFP), and GAPDH in *Erk* DKO ESC lines carrying the indicated ERK mutants or RFP protein under basal medium plus LIF condition. WT mouse ES cells were used as the control.

(C) qPCR analysis of *Egr1* expression in *Erk* DKO ESC lines expressing the control (RFP), *Erk1 WT*, or *Erk2 WT* under indicated conditions. Cells were starved in serum free medium overnight and then treated with serum, serum + 1μM PD03, serum + 1μM Vx11e for 3 hours before qPCR analysis. Data represent mean ± SEM (n=3). ***: $p<0.001$.

(D) Western blot analysis of the expression of phosphorylated RSK (pRSK), total RSK (tRSK), phosphorylated ERK (pERK), total ERK (tERK), and GAPDH in *Erk* DKO ESCs expressing the control (RFP), *Erk1 WT*, or *Erk2* WT under indicated conditions. Cells were treated with the indicated combination of bFGF, PD03 and Vx11e for 10-15 min after overnight serum starvation.

(E) ESC self-renewal and differentiation can be used as the read-out for the kinase activity of ERK proteins. Representative phase contrast images of *Erk* DKO ESC lines expressing the control (RFP), *Erk1 WT*, or *Erk2 WT* cultured under indicated conditions in N2B27 medium for 7 days. Scale bars, 100μm.

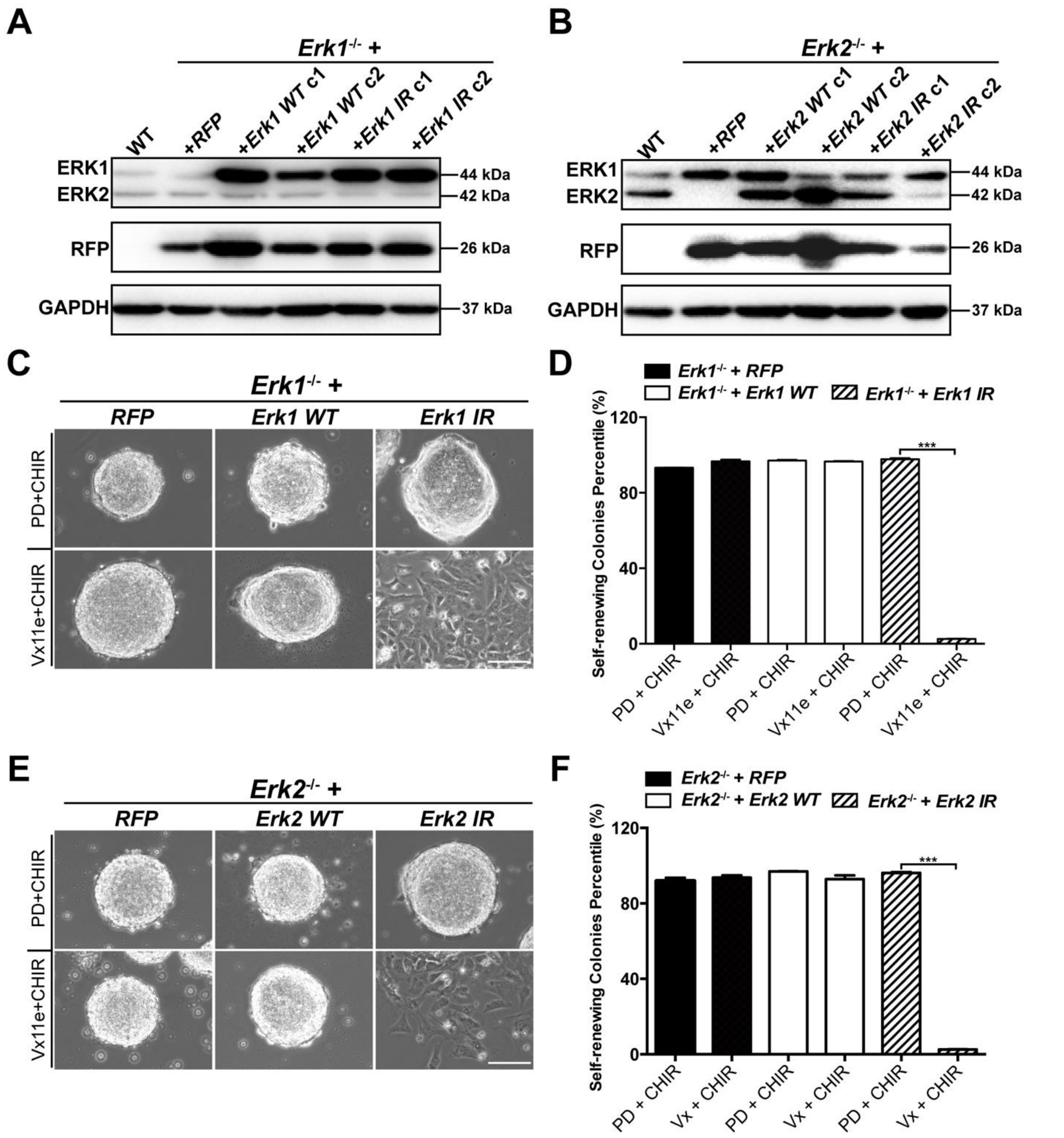


**Figure S3. Selective inhibition of one ERK paralog is not sufficient to maintain mouse ESC self-renewal.**

(A-B) Western blot analysis of the expression of ERK1/2, RFP, and GAPDH in *Erk1*$^{-/-}$ ESC lines carrying the indicated ERK1 mutants or RFP protein (A) and in *Erk2*$^{-/-}$ ESC lines carrying the indicated ERK2 mutants or RFP protein (B) under basal medium plus LIF condition. The WT mouse ESCs were used as control.

(C) Representative phase contrast images of *Erk1*$^{-/-}$ ESCs expressing RFP (control), ERK1 WT, or ERK1 IR mutant under indicated conditions in N2B27 medium for 7 days. Scale bars, 100μm.

(D) Quantification of the percentage of self-renewing colonies (AP staining positive) in *Erk1*$^{-/-}$ ESCs expressing RFP (control), ERK1 WT, or ERK1 IR mutant cultured in N2B27 medium supplemented with PD + CHIR, Vx11e + CHIR, or CHIR. Data represent means ± SEM (n=2). ***: $p < 0.001$.

(E) Representative phase contrast images of *Erk2*$^{-/-}$ ESCs expressing RFP (control), ERK2 WT, or ERK2 IR mutant under indicated conditions in N2B27 medium for 7 days. Scale bars, 100μm.

(F) Quantification of the percentage of self-renewing colonies (AP staining positive) in Erk2$^{-/-}$ ESCs expressing RFP control, ERK2 WT, or ERK2 IR mutant cultured in N2B27 medium supplemented with PD + CHIR, Vx11e + CHIR, or CHIR. Data represent means ± SEM (n=2). ***: $p < 0.001$.

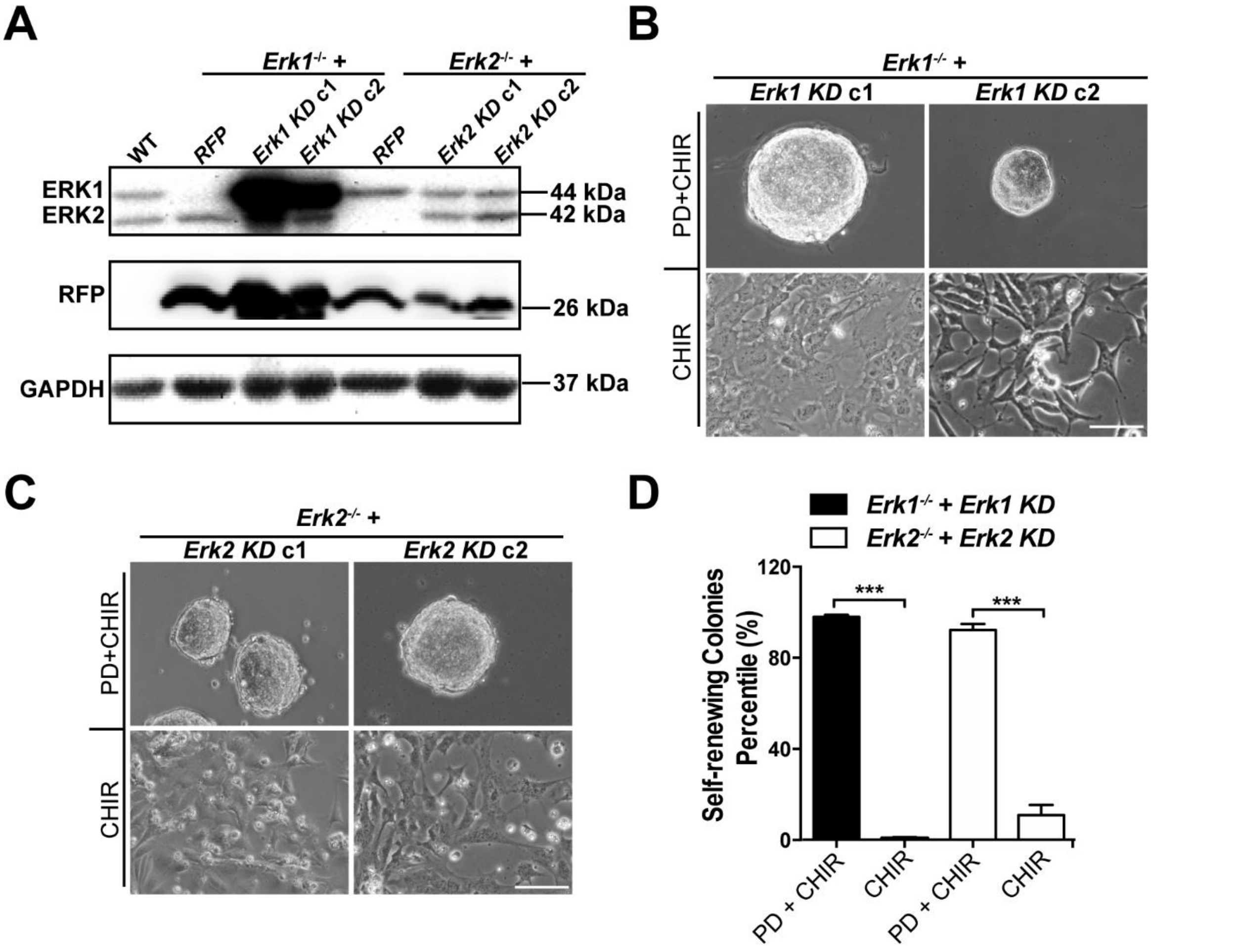


**Figure S4. Selective inhibition of ERK paralogs through a kinase-dead approach cannot maintain ESC self-renewal in the presence of CHIR.**

(A) Western blot analysis of the expression of ERK1/2, RFP, and GAPDH in WT ESCs, *Erk1*$^{-/-}$ ESCs expressing RFP or ERK1 KD mutant, and *Erk2*$^{-/-}$ ESCs expressing RFP or ERK2 KD mutant.

(B-C) Representative phase contrast images of *Erk1*$^{-/-}$ ESCs expressing ERK1 KD (B), and *Erk2*$^{-/-}$ ESCs expressing ERK2 KD (C) in N2B27 medium supplemented with PD + CHIR or CHIR for 7 days. Scale bar, 100μm.

(D) Quantification of the percentage of self-renewing colonies formed using AP staining in *Erk1*$^{-/-}$ ESCs expressing ERK1 KD mutant, *Erk2*$^{-/-}$ ESCs expressing ERK2 KD mutant cultured in N2B27 medium supplemented with PD + CHIR or CHIR. Data represent means ± SEM (n=3). ***: $p < 0.001$.

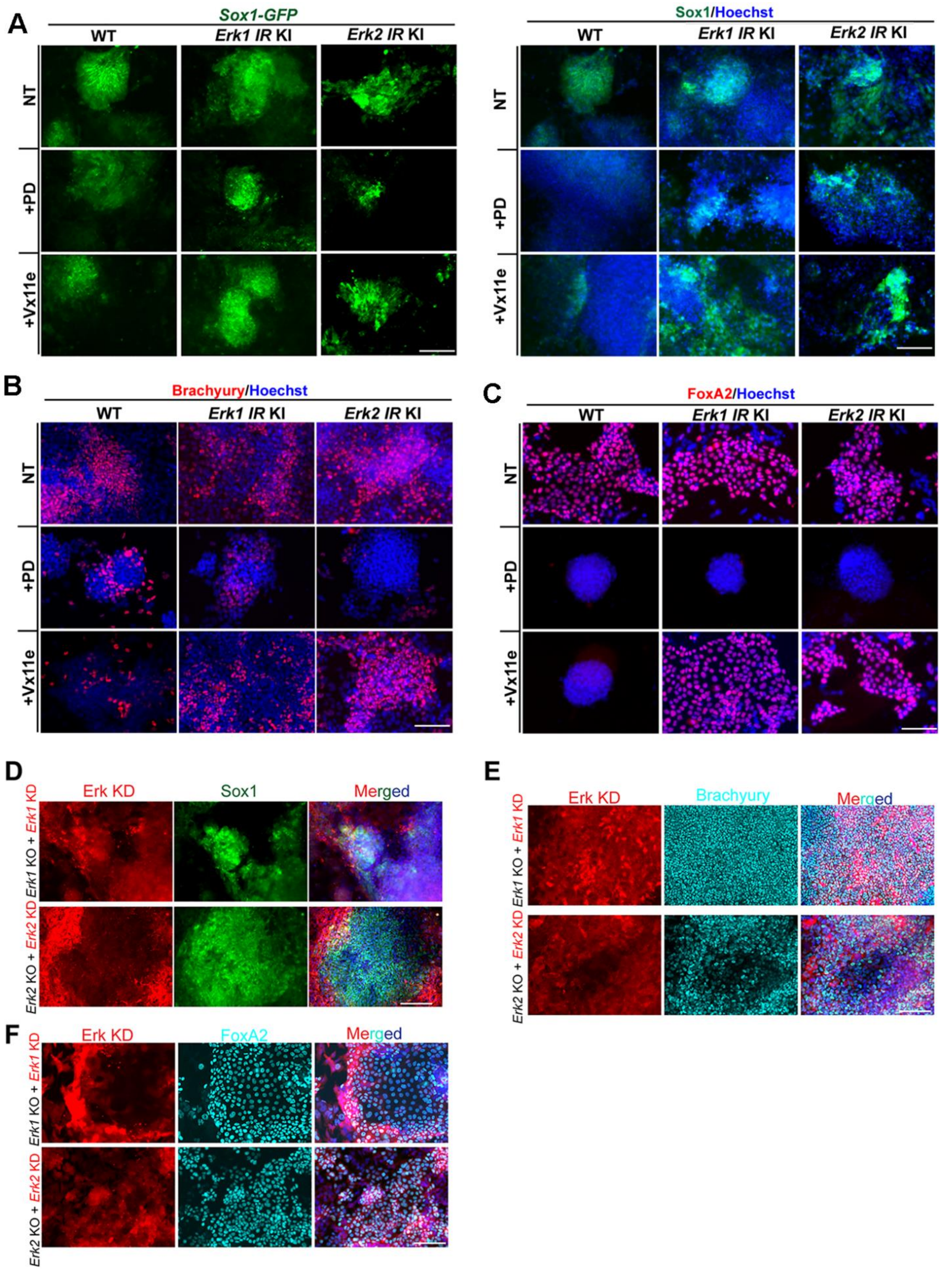
A
Sox1-GFP
WT
Erk1 IR KI
Erk2 IR KI
NT
+PD
+Vx11e
Sox1/Hoechst
B
Brachyury/Hoechst
C
FoxA2/Hoechst
D
Erk KD
Sox1
Merged
Erk1 KO + Erk1 KD
Erk2 KO + Erk2 KD
E
Brachyury
F
FoxA2

**Figure S5. Inhibition of either ERK1 or ERK2 cannot block ESC differentiation.**

(A) Representative immunofluorescent staining images of SOX1-EGFP expression in WT ESCs, *Erk1* IR KI ESCs, and *Erk2* IR KI ESCs differentiating in N2B27 medium under the indicated conditions for 6 days. Scale bars, 100μm.

(B) Representative immunofluorescent staining images of Brachyury (Red) in WT ESCs, *Erk1* IR KI ESCs, and *Erk2* IR KI ESCs differentiating in mesoderm induction medium under the indicated conditions for 6 days. Scale bars, 100μm.

(C) Representative immunofluorescent staining images of FoxA2 (Red) in WT ESCs, *Erk1* IR KI ESCs, and *Erk2* IR KI ESCs differentiating in endoderm induction medium under the indicated conditions for 7 days. Scale bars, 100μm.

(D-F) Representative immunofluorescent staining images of SOX1 (Green), Brachyury (Cy5), and FoxA2 (Cy5) in *Erk1*$^{-/-}$ + *Erk1 KD* ESCs and *Erk2*$^{-/-}$ + *Erk2 KD* ESCs undergoing differentiation. Scale bars, 100μm.
